# Structure of apo flavin-dependent halogenase Xcc4156 hints at a reason for cofactor-soaking difficulties

**DOI:** 10.1107/S2059798320007731

**Published:** 2020-06-30

**Authors:** Christiane Widmann, Mohamed Ismail, Norbert Sewald, Hartmut H. Niemann

**Affiliations:** a Structural Biochemistry (BCIV), Department of Chemistry, Bielefeld University, Universitaetsstrasse 25, 33615 Bielefeld, Germany; b Organic and Bioorganic Chemistry (OC III), Department of Chemistry, Bielefeld University, Universitaetsstrasse 25, 33615 Bielefeld, Germany

**Keywords:** FAD-dependent halogenase, crystal soaking, cofactor binding, enzymatic halogenation, bromination, crystal structure

## Abstract

Flavin-dependent halogenases regioselectively introduce halide substituents into electron-rich substrates under mild reaction conditions. For the enzyme Xcc4156 from *Xanthomonas campestris*, the structure of a complex with the cofactor flavin adenine dinucleotide (FAD) and a bromide ion would be of particular interest as this enzyme exclusively brominates model substrates *in vitro*. Apo Xcc4156 crystals diffracted to 1.6 Å resolution. The structure revealed an open substrate-binding site lacking the loop regions that close off the active site and contribute to substrate binding in tryptophan halogenases. Therefore, Xcc4156 might accept larger substrates, possibly even peptides. Soaking of apo Xcc4156 crystals with FAD led to crumbling of the intergrown crystals. Around half of the crystals soaked with FAD did not diffract, while in the others there was no electron density for FAD. The FAD-binding loop, which changes its conformation between the apo and the FAD-bound form in related enzymes, is involved in a crystal contact in the apo Xcc4156 crystals. The conformational change that is predicted to occur upon FAD binding would disrupt this crystal contact, providing a likely explanation for the destruction of the apo crystals in the presence of FAD. Soaking with only bromide did not result in bromide bound to the catalytic halide-binding site. Simultaneous soaking with FAD and bromide damaged the crystals more severely than soaking with only FAD. Together, these latter two observations suggest that FAD and bromide bind to Xcc4156 with positive cooperativity. Thus, apo Xcc4156 crystals provide functional insight into FAD and bromide binding, even though neither the cofactor nor the halide is visible in the structure.

## Abstract 2

Xcc4156 is a flavin-dependent halogenase from *Xanthomonas campestris*. Here, an apo structure is presented which, in combination with soaking experiments, provides functional insight into the binding of FAD and bromide to the halogenase.

## 1. Introduction   

The activation of C—H bonds in electron-rich organic compounds is an important step that enables their use in a range of downstream applications such as metal-catalyzed cross-coupling reactions or modifications via nucleophilic substitution (Runguphan & O’Connor, 2013 ▸; Frese *et al.*, 2016 ▸; Durak *et al.*, 2016 ▸). Chemical halogenation requires harsh conditions, often employing elemental halogens in combination with Lewis acids. In addition, the process may lead to the formation of byproducts owing to a lack of regioselectivity. Flavin-dependent halogenases (FDHs) have come into focus as a clean alternative because they halogenate their substrates under much milder conditions using halide salts and molecular oxygen at 25°C and pH 7 (Keller *et al.*, 2000 ▸; Yeh *et al.*, 2005 ▸). Their reaction mechanism has been investigated both *in vitro* (Dong *et al.*, 2005 ▸; Yeh *et al.*, 2006 ▸, 2007 ▸; Flecks *et al.*, 2008 ▸; Fraley *et al.*, 2017 ▸) and *in silico* (Fraley *et al.*, 2017 ▸; Ainsley *et al.*, 2018 ▸). FDHs depend on reduced flavin adenine dinucleotide (FADH_2_) as a cofactor, which is bound by a dedicated subdomain. A halide ion (X^−^) is coordinated close to the isoalloxazine ring of FAD (Dong *et al.*, 2005 ▸). As a first reaction step, FAD-C4a-OOH is formed (Yeh *et al.*, 2006 ▸), which subsequently oxidizes the halide ion, resulting in the formation of hypohalous acid (HOX). The HOX then travels around 10 Å to the substrate-binding site of the enzyme, where halogenation takes place (Dong *et al.*, 2005 ▸). A conserved active-site lysine plays a crucial role in the halogenation, either via hydrogen bonding to HOX (Flecks *et al.*, 2008 ▸) or by forming a lysine haloamine (Yeh *et al.*, 2007 ▸). The exact mechanism is still under debate, but the electrophilic aromatic substitution takes place at the substrate C atom that is closest to the catalytic lysine and, owing to substrate positioning by the enzyme, the reaction is regio­selective.

The first FDHs to be characterized were tryptophan halogenases (Trp halogenases; Keller *et al.*, 2000 ▸; Yeh *et al.*, 2005 ▸). In the following years, a variety of different halogenases were studied that can be grouped into indole halogenases (which include the Trp halogenases; Seibold *et al.*, 2006 ▸; Heemstra & Walsh, 2008 ▸; Zeng & Zhan, 2011 ▸; Menon *et al.*, 2016 ▸; Ma *et al.*, 2017 ▸; Lingkon & Bellizzi, 2020 ▸; Domergue *et al.*, 2019 ▸; Luhavaya *et al.*, 2019 ▸), phenol halogenases (Buedenbender *et al.*, 2009 ▸; Neumann *et al.*, 2010 ▸; Zeng *et al.*, 2013 ▸; Agarwal *et al.*, 2014 ▸; Menon *et al.*, 2017 ▸; Mori *et al.*, 2019 ▸) and pyrrole halogenases (Hammer *et al.*, 1997 ▸; Dorrestein *et al.*, 2005 ▸; Yamanaka *et al.*, 2012 ▸; Agarwal *et al.*, 2014 ▸), depending on the classes of compounds that they halogenate.

Most described indole halogenases belong to the subgroup of Trp halogenases. Using genomic data, we recently identified four halogenases that act on indole derivatives but not on free tryptophan. The native substrates of BrvH, a halogenase identified from a marine metagenome (Neubauer *et al.*, 2018 ▸), as well as of three halogenases from *Xanthomonas campestris* pv. *campestris* B100 (Ismail *et al.*, 2019 ▸), remain unknown. While BrvH preferentially brominated screening substrates, the *X. campestris* (Xcc) halogenases exclusively brominated them. Previously, only Bmp2 and Bmp5, two marine halogenases from *Pseudoalteromonas* spp., had been shown to selectively incorporate bromide into their substrates (Agarwal *et al.*, 2014 ▸). Bmp2 is responsible for the tribromination or tetrabromination of a carrier-tethered pyrrole (El Gamal *et al.*, 2016 ▸), whereas Bmp5 catalyzes the bromination and decarboxylation of 4-hydroxybenzoic acid (Agarwal *et al.*, 2014 ▸). Both enzymes do not catalyze chlorination, but for Bmp5 *in vivo* iodination was additionally observed. The exclusive bromination by Bmp2 was attributed to halide specificity (Thapa *et al.*, 2018 ▸). A crystal structure of Bmp2 lacked bound halide and did not reveal the molecular basis of the specificity for bromide (El Gamal *et al.*, 2016 ▸). In contrast to brominases that do not chlorinate, several FDHs that natively chlorinate their substrates can also catalyze bromination. Thal and Mpy16, for example, which chlorinate tryptophan and pyrrole, respectively, form brominated products if an excess of bromide is present (Seibold *et al.*, 2006 ▸; Thapa *et al.*, 2018 ▸). RebH, a Trp halogenase that mediates the chlorination step during rebeccamycin biosynthesis, has been shown to bromin­ate a larger number of non-natural probe substrates than it can chlorinate, leading Lewis and coworkers to suggest that owing to the higher electrophilicity of bromine relative to chlorine in hypohalous acid, a preference for bromination is common in FDHs (Fisher *et al.*, 2019 ▸).

We set out to investigate whether the halide specificity of brominases can be attributed to the structural determinants of bromide binding. To this end, we aimed at elucidating the structure of Xcc4156, one of the three brominating Xcc halogenases, in complex with its cofactor and bromide.

## 2. Methods   

### 2.1. Protein expression   

The *xcc4156* gene was amplified from a pET-28a plasmid (Ismail *et al.*, 2019 ▸) and cloned into a pETM-11 expression vector which contains a hexahistidine tag followed by a tobacco etch virus (TEV) protease cleavage site. This plasmid was transformed into *Escherichia coli* BL21 (DE3) pGro7 cells. The pGro7 plasmid (Takara) codes for the expression of the GroEL–GroES chaperone system.

An overnight preculture (30°C, 110 rev min^−1^) was used to inoculate a 2 l culture in LB medium containing 30 µg ml^−1^ kanamycin and 34 µg ml^−1^ chloramphenicol to an optical density at 600 nm (OD_600_) of 0.1. The culture was incubated at 37°C (90 rev min^−1^) until an OD_600_ of 0.4 was reached. 2 g l^−1^ l-arabinose and 0.1 m*M* isopropyl β-d-1-thiogalactopyranoside were added for the induction of chaperone and target protein expression, and the cultivation temperature was decreased to 25°C. The cells were harvested by centrifugation after 20 h and the resulting cell pellets were washed with phosphate-buffered saline (PBS) and frozen at −20°C.

### 2.2. Protein purification   

The purification protocol included an immobilized metal-affinity chromatography (IMAC) step followed by ion-exchange (IEX) chromatography, TEV digestion and another IMAC step. The exact protocol has been described in Neubauer *et al.* (2018 ▸) and was followed except for the size-exclusion chromatography step. Protein obtained from the flowthrough and washing steps of the second IMAC step was concentrated using a Vivaspin column (10 000 Da molecular-weight cutoff; Sartorius, Germany) to 29.19 mg ml^−1^ and was stored at −80°C prior to protein characterization and crystallization.

### 2.3. Protein characterization   

To analyze whether the protein forms a monomer or a dimer in solution, dynamic light scattering (DLS) as well as size-exclusion chromatography (SEC) were performed.

For DLS, Xcc4156 was diluted to 5 mg ml^−1^ with 10 m*M* Tris, 150 m*M* NaCl pH 8. Samples were centrifuged at 30 000*g* for 30 min prior to analysis and were transferred to a quartz glass cuvette with a path length of 1.5 mm. Measurements were performed using a DynaPro 801 and the molecular weight was calculated from the hydrodynamic radius using the *DYNAMICS* software (version 5.25.44). The DLS experiment was performed in triplicate. The calculated percentage polydispersities were 8%, 5.42% and 7.77% and the calculated molecular masses were 97.0, 94.9 and 97.7 kDa.

For gel-filtration chromatography, Xcc4156 was diluted to 4, 0.4 and 0.04 mg ml^−1^ using 50 m*M* Tris, 150 m*M* NaCl pH 8. The same buffer was used for isocratic elution. 100 µl protein solution was applied onto a Superdex 200 10/300 column with a bed volume of ∼21 ml. The molecular weight was obtained following calibration with ferritin (440 kDa), conalbumin (75 kDa), ovalbumin (44 kDa) and RNase A (13.7 kDa). Peak detection was performed in *UNICORN* (version 5.20). The obtained molecular masses were 105, 105 and 102 kDa for the samples at 4, 0.4 and 0.04 mg ml^−1^, respectively.

### 2.4. Protein crystallization and soaking   

For crystallization of the apo protein, Xcc4156 was diluted to 10 mg ml^−1^ with 10 m*M* Tris, 150 m*M* NaCl pH 8. Crystallization conditions were screened using The JCSG Core IV Suite (Qiagen, Germany) in MRC 2 Lens Plates (SWISSCI, Switzerland). A bunch of thin needles appeared within one day at 20°C in 1 *M* sodium/potassium tartrate, 0.1 *M* MES pH 6.0 using a drop size of 0.3 µl and a protein:reservoir solution ratio of 2:1. Optimization (0.6 *M* sodium/potassium tartrate, 0.1 *M* MES pH 6.5; drop volume 3 µl) yielded thicker needles, but the crystals remained intergrown. Prior to data collection, the protruding part of a large needle was briefly transferred into a solution consisting of 0.7 *M* sodium/potassium tartrate, 0.1 *M* MES pH 6.5, 17.5% l-2,3-butanediol and flash-cooled in liquid nitrogen.

For soaking, another optimization plate (0.6 *M* sodium/potassium tartrate, 0.1 *M* MES pH 6.0) was set up with a drop size of 1.5 µl containing protein and reservoir solution in the same ratio as before. When the crystals had grown (usually within a week), an additional 0.5 µl of ligand solution [‘FAD’ (0.6 *M* sodium/potassium tartrate, 0.1 *M* MES pH 6.0, 15 m*M* FAD), ‘NaBr’ (0.6 *M* sodium/potassium tartrate, 0.1 *M* MES pH 6.0, 150 m*M* NaBr), ‘FAD–NaBr’ (0.6 *M* sodium/potassium tartrate, 0.1 *M* MES pH 6.0, 15 m*M* FAD, 150 m*M* NaBr) or ‘NaBr200’ (0.6 *M* sodium/potassium tartrate, 0.1 *M* MES pH 6.0, 200 m*M* NaBr)] was added to the drop. Incubation was performed for one day. Photographs were taken after 0 and 24 h. Crystals were harvested after 24 h of soaking following the protocol used for the apo crystals but with the ligands present in the cryosolution at the same concentration as in the ligand solution.

### 2.5. Protein structure determination   

Measurements were carried out on the BL14.2 beamline at Helmholtz-Zentrum Berlin (Gerlach *et al.*, 2016 ▸). For both apo and soaked Xcc4156 crystals, 3600 frames were collected with an oscillation angle of 0.1°. The X-ray wavelength was 0.917143 Å for the apo crystals and 0.9184 Å for the soaked crystals. The raw data were processed with *XDS* and scaled with *XSCALE* (Kabsch, 2010 ▸). For the apo data set, the data were imported into *CCP*4 (*CCP*4*i*2 interface; Potterton *et al.*, 2018 ▸) and merged [cut at *I*/σ(*I*) = 2] with *AIMLESS* (Evans & Murshudov, 2013 ▸) for structure solution via molecular replace­ment using *MrBUMP* (Keegan & Winn, 2007 ▸; McCoy *et al.*, 2007 ▸); the best search model was chain *A* of BrvH (PDB entry 6frl), with a sequence identity of 45.5%. An initial model was built by *Buccaneer* (Cowtan, 2006 ▸). Manual model building was performed in *Coot* (Emsley *et al.*, 2010 ▸), and both *REFMAC*5 (Murshudov *et al.*, 2011 ▸) and *phenix.refine* (Liebschner *et al.*, 2019 ▸) were used for crystallographic refinement of the interim models. Restrained refinement was used and *B* factors were considered to be isotropic. Non­crystallographic symmetry (NCS) was used to generate further restraints. Validation with *MolProbity* (Chen *et al.*, 2010 ▸) was part of the building process. The statistics of the final model are given in Table 1 ▸, and the coordinates and structure factors have been deposited in the Protein Data Bank (PDB entry 6y1w).

Data processing of data sets from the soaked crystals was performed similarly to the apo data set, but as we were looking for anomalous signal, Friedel mates were treated as different reflections for soaks containing NaBr. As the soaked and apo crystals had very similar unit-cell parameters, rigid-body refinement of the apo model against the respective data followed by three cycles of restrained refinement was performed in *phenix.refine*. The apo model was used to generate additional restraints. The resulting electron-density maps were evaluated in *Coot* with a focus on the FAD-binding and halide-binding sites. No further model building was performed, and the structures were not deposited in the PDB. The statistics of the models are given in Table 1 ▸. Figures were generated in *PyMOL*.

### 2.6. Bioinformatics   

A multiple sequence alignment was performed in *Clustal Omega* (Madeira *et al.*, 2019 ▸) using the default settings. 44 FDH sequences (given in the supporting information) were input and a phylogenetic tree was calculated from the resulting alignment. The tree was calculated in *Jalview* 2.10.5 (Waterhouse *et al.*, 2009 ▸) using the neighbour-joining algorithm and the BLOSUM62 matrix. The structures of the most similar halogenases (Table 2 ▸) and halogenases that clustered more distantly with the Trp halogenases were additionally compared with the Xcc4156 structure using the *DALI* server (Holm, 2019 ▸) in an ‘all-against-all structure comparison’. The resulting comparison was uploaded to *ClustalW*2 (Madeira *et al.*, 2019 ▸) in order to generate a phylogenetic tree using the neighbour-joining algorithm and distance correction including gaps.

In addition, a *DALI* comparison (Holm, 2019 ▸) of the structures of Xcc4156 (PDB entry 6y1w chain *A*), BrvH (PDB entry 6frl chain *A*; Neubauer *et al.*, 2018 ▸), Th-Hal (PDB entry 5lv9 chain *B*; Menon *et al.*, 2016 ▸) and RebH (PDB entry 2oal chain *B*; Yeh *et al.*, 2007 ▸) was performed and used as input for the *ESPript* 3.0 server (Robert & Gouet, 2014 ▸). The alignment was also used in Fig. 2, in which the conservation of the amino acids at each position is shown. This quantitative annotation was output by *Jalview* (Waterhouse *et al.*, 2009 ▸) based on the number of conserved physicochemical properties. Its calculation is based on that used in the *AMAS* method of multiple sequence alignment analysis (Livingstone & Barton, 1993 ▸).

In order to compare the crystal contacts that the loops of RebH (PDB entry 2oam; Yeh *et al.*, 2007 ▸) and PltM (PDB entry 6bzn; Mori *et al.*, 2019 ▸) as well as Xcc4156 are involved in, the *PISA* server (Krissinel & Henrick, 2007 ▸) was used.

## 3. Results   

### 3.1. Crystal structure of Xcc4156   

Our attempts to co-crystallize Xcc4156 with FAD and bromide failed. A total of 28 96-well plates were set up with different commercial screens, at different protein concentrations and at different temperatures (see Supplementary Table S2). No diffracting crystals were obtained. However, apo Xcc4156 yielded crystals that diffracted to a resolution of 1.6 Å (see Table 1 ▸).

**Figure 1:**
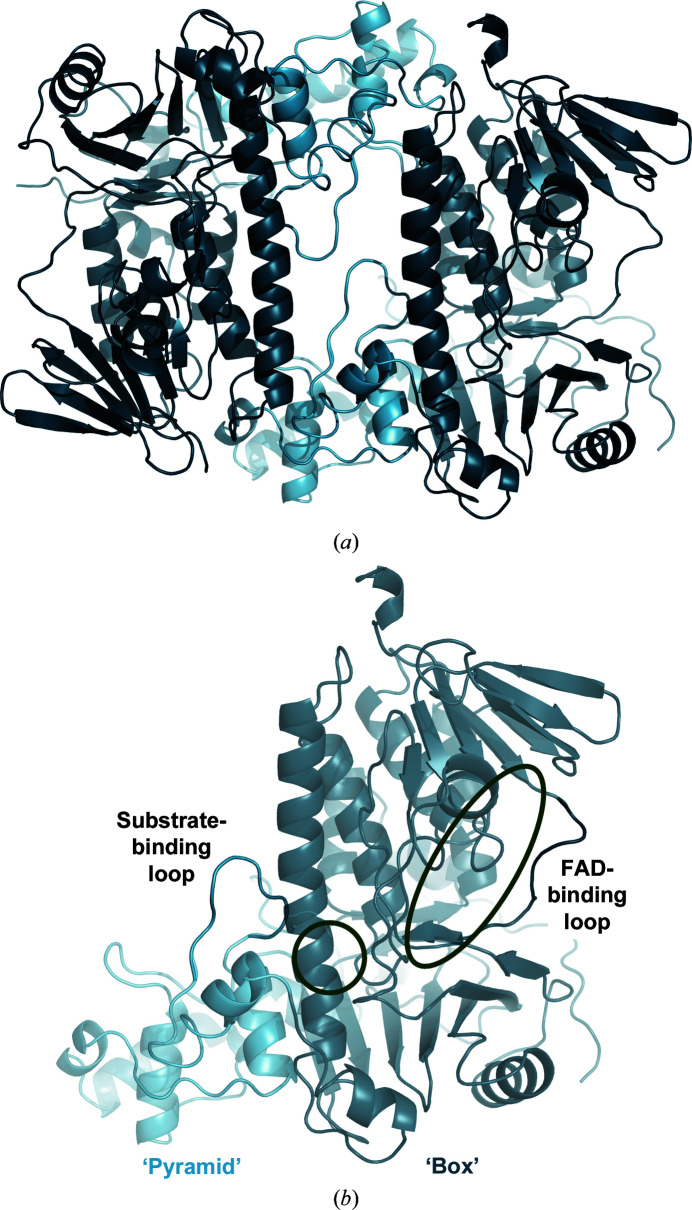
Crystal structure of Xcc4156. (*a*) Dimer in the crystal of Xcc4156 (PDB entry 6y1w). The structurally conserved part between different FDHs (‘box’) is shown in dark blue and the structurally variable ‘pyramid’ is shown in light blue. (*b*) Monomer of Xcc4156 (chain *A*). The FAD-binding loop in the ‘box’ subdomain and a loop at the substrate-binding site at the interface between the ‘box’ and the ‘pyramid’ subdomains are highlighted. The inferred FAD-binding and substrate-binding sites (deduced from their respective positions in Trp halogenases) are marked as green ovals.

In the crystal, there are two chains in the asymmetric unit, with only minor differences between them (Fig. 1 ▸ *a*). Each of these is a halogenase monomer consisting of two subdomains: a ‘box’ subdomain containing the FAD-binding site, which is structurally conserved among FDHs, and a more variable ‘pyramid’ subdomain (Fig. 1 ▸ *b*). The substrate-binding site of FDHs is at the interface between the box and pyramid sub­domains (Dong *et al.*, 2005 ▸). The dimer observed in the crystal is very similar to the crystallographic dimers of Trp halogenases (Dong *et al.*, 2005 ▸; Yeh *et al.*, 2007 ▸; Zhu *et al.*, 2009 ▸; Shepherd *et al.*, 2016 ▸; Menon *et al.*, 2016 ▸; Moritzer *et al.*, 2019 ▸; Luhavaya *et al.*, 2019 ▸; Lingkon & Bellizzi, 2020 ▸). Estimates of the molecular weight from the hydrodynamic radius obtained by size-exclusion chromatography (SEC) and dynamic light scattering (DLS) are 105 and 96.5 ± 1.5 kDa, respectively. These values are substantially higher than that of a monomer (58.1 kDa), but are still lower than the molecular weight of a dimer (116.2 kDa). SEC was performed on samples between 4 and 0.04 mg ml^−1^, giving very similar results. This renders a concentration-dependent change in the oligomerization state very unlikely. As a nonglobular shape can explain an apparent molecular weight that is higher but not lower than the actual value, we take the SEC and DLS results as an indication that Xcc4156 may be monomeric in solution. In Thal, a tryptophan halogenase, SEC and DLS also hinted towards a monomer, as is the case here, but the enzyme likewise formed a dimer in the crystal (Moritzer *et al.*, 2019 ▸). ESI-MS under native conditions revealed that Thal was present both as a homodimer and a monomer in solution (Minges *et al.*, 2020 ▸). The same might be the case for Xcc4156. The presence of the same dimer in all Trp halogenase crystals, and in solution in the case of PrnA as observed by SEC (Dong *et al.*, 2005 ▸), suggests an evolutionary advantage of this association, which is supported by a study on Thal mutants in which increased thermostability coincided with a higher proportion of the protein being present as a homodimer in solution (Minges *et al.*, 2020 ▸).

To further investigate the similarity to known halogenases, a multiple sequence alignment of 44 FDH sequences was performed in *Clustal Omega* (Madeira *et al.*, 2019 ▸). Additionally, the Xcc4156 structure was compared with the structures of selected halogenases (Table 2 ▸) using the *DALI* server (Holm, 2019 ▸) and a phylogenetic tree was created from the *DALI* comparison (Supplementary Fig. S1).

The sequence alignment and structural comparison performed in this study confirmed the high similarity of Xcc4156 to Trp halogenases that we expected based on previous sequence alignments (Ismail *et al.*, 2019 ▸). In accordance, Xcc4156 shares their overall structure (see above). Xcc4156 has 45% sequence identity to BrvH (Neubauer *et al.*, 2018 ▸) and around 30–35% sequence identity to various Trp halogenases (for example 34% to both PyrH and RebH and 32% to PrnA).

**Figure 2:**
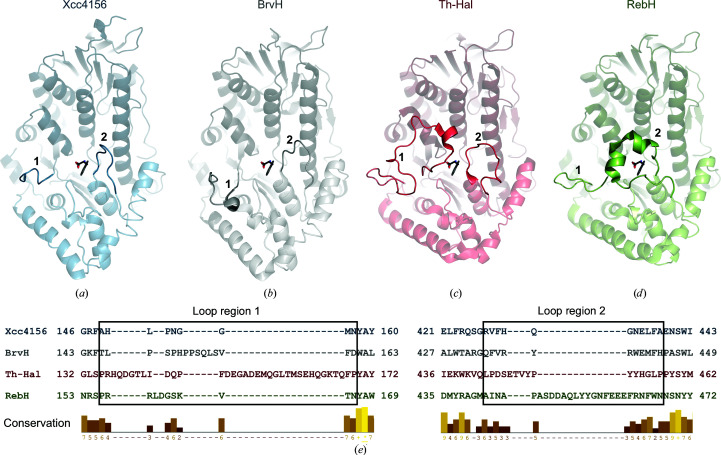
Structural comparison of Xcc4156 with BrvH, Th-Hal and RebH. (*a*) Xcc4156 chain *A* (blue). (*b*) BrvH (grey; PDB entry 6frl chain *A*). (*c*) Th-Hal (red; PDB entry 5lv9 chain *B*). (*d*) RebH (green; PDB entry 2oa1 chain *A*). In (*a*)–(*d*) the tryptophan from RebH (grey) is shown to mark the location of the substrate-binding site, as neither the Xcc4156 structure nor the BrvH and Th-Hal structures contain a bound substrate. Two loop regions are highlighted that form part of the substrate-binding site in two clades of halogenases. Region ‘1’ is the substrate-binding loop of the C5 Trp halogenase group containing Th-Hal (amino acids 149–157, 146–160, 135–169 and 156–166 in Xcc4156, BrvH, Th-Hal and RebH, respectively). Region ‘2’ is the substrate-binding loop of the C7 Trp halogenase group that RebH belongs to (amino acids 428–438, 434–444, 443–457 and 442–467 in Xcc4156, BrvH, Th-Hal and RebH, respectively). Xcc4156 and BrvH have a short loop in both positions, resulting in a very open substrate-binding site. Refer to Supplementary Fig. S1 for a phylogenetic tree. (*e*) Alignment based on a *DALI* structure comparison showing the sequences of Xcc4156, BrvH, Th-Hal and RebH in both loop regions. The rectangle encloses the loop regions that are highlighted in (*a*)–(*d*). The conservation of the amino acids (yellow) was output by *Jalview*.

As expected, based on the high sequence identity, the structure of Xcc4156 resembles that of BrvH (Figs. 2 ▸ *a* and 2 ▸ *b*). Their substrate-binding region distinguishes Xcc4156 and BrvH from the Trp halogenases. When clustered after a *BLAST* pairwise alignment, the latter form two groups (Fisher *et al.*, 2019 ▸). In addition to other minor differences, particularly in the loops, the substrate coordination differs between these groups (Figs. 2 ▸ *a*–2 ▸ *d*; Luhavaya *et al.*, 2019 ▸). One group, containing, for example, SttH, Th-Hal, PyrH, KtzR and Tar14, has a long substrate-binding loop (‘1’ in Fig. 2 ▸ *c*). This group includes C5 Trp halogenases, which halogenate their substrate tryptophan in the C5 position, as well as some C6 Trp halogenases. The substrate-binding loop of the second group, which contains, for example, RebH, Thal, PrnA and KtzQ, is shorter and connects to a different side of the pyramid (‘2’ in Fig. 2 ▸ *d*). This group comprises all known C7 Trp halogenases as well as some C6 Trp halogenases.

Xcc4156 and BrvH have short loops in both positions, resulting in a more open substrate-binding site compared with Trp halogenases and a solvent-accessible catalytic lysine residue. In Trp halogenases the loop usually closes over the substrate upon binding. As Xcc4156 and BrvH have much shorter loops, this seems very unlikely in these two enzymes. The absence of the substrate-binding loop is reflected in the residues adjacent to the substrate-binding site. Whereas the catalytic residues in RebH all have corresponding and similar residues in Xcc4156 (refer to Supplementary Table S1) and a part of the π-stacking region is also similar, the backbone binding region of RebH does not have a counterpart in Xcc4156. Like BrvH, Xcc4156 does not halogenate free tryptophan (Neubauer *et al.*, 2018 ▸; Ismail *et al.*, 2019 ▸); these enzymes do, however, show activity towards free indole and indole derivatives. This can be explained by the lack of stabilization of tryptophan in the substrate-binding site. Thus, the hypothesis that BrvH might halogenate a peptide (Neubauer *et al.*, 2018 ▸), like MibH (Ortega *et al.*, 2017 ▸), also applies to Xcc4156. A peptide or larger tryptophan derivative could interact with the halogenase surface, orienting the substrate in the active site. It should be noted, however, that Xcc4156 only shares 26% sequence identity with MibH, and both Xcc4156 and BrvH do not cluster close to MibH based on sequence alignment (data not shown), an observation that was also made by Fisher *et al.* (2019 ▸).

**Figure 3:**
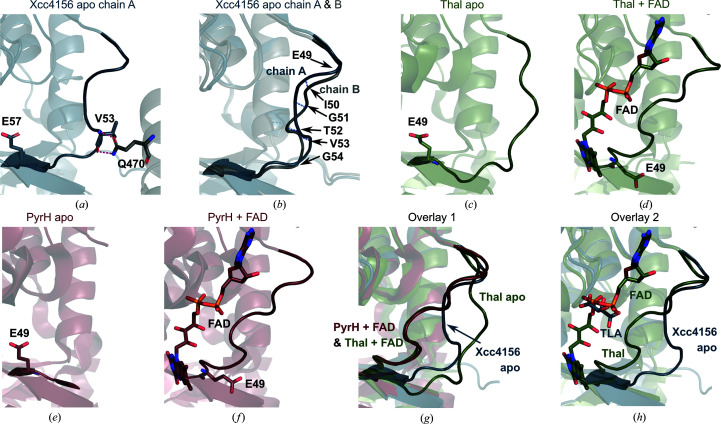
Comparison of the FAD-binding loop of Xcc4156 with the open and closed FAD-binding loops of Thal and PyrH. (*a*) The crystal contact that fixes the loop in chain *A* (blue; amino acids 47–58) of Xcc4156 in position. The backbone atoms of Val53 in chain *A* form two hydrogen bonds (pink) to the Gln470 side-chain amide in chain *B* of a symmetry-related molecule (grey). (*b*) Overlay of chain *A* of Xcc4156 (blue) with chain *B* (grey). Light blue dashed lines connect the same amino acids in both chains. The distances between C^α^ atoms are 0.8, 1.7, 2.5, 2.9, 2.6 and 1.0 Å for Glu49–Gly54. (*c*) The FAD-binding loop of Thal (green; PDB entry 6h43 chain *A*; amino acids 39–50) in the ‘open’ conformation when no FAD is bound. (*d*) Upon FAD binding, the FAD-binding loop closes (PDB entry 6sls chain *A*). (*e*) In the absence of FAD, the FAD-binding loop of PyrH could not be modelled (red; PDB entry 2weu; amino acids 36–47). (*f*) When FAD is bound the loop closes, taking on the same conformation as in Thal (PDB entry 2wet chain *A*). In (*a*), (*c*), (*d*), (*e*) and (*f*), a conserved glutamate residue (Glu57 in Xcc4156 and Glu49 in both Thal and PyrH) is shown which acts as a sensor for FAD binding. (*g*) Overlay of apo Xcc4156 chain *A* (blue), apo Thal (green), Thal + FAD (green) and PyrH + FAD (red). Loops are open in the apo state and closed in the FAD-bound state. (*h*) Overlay of apo Xcc4156 chain *A* (blue) and Thal + FAD (green). In Xcc4156, an l-tartaric acid (TLA) molecule is bound at the site that coordinates a phosphate of FAD in other halogenases.

The FAD-binding loop (amino acids 47–56) of Xcc4156 does not have continuous electron density in chain *B* (Supplementary Fig. S4*b*). Its position seems to be mainly stabilized by a weak interaction between the carbonyl O atom of Gly51 and a hydrogen of the guanidino group of Arg313 (both in chain *B*). In chain *A*, however, the loop is well defined as it is part of a crystal contact that is stabilized by two hydrogen bonds between the backbone N and O atoms of Val53 in chain *A* and the side-chain amide of Gln470 in chain *B* (Figs. 3 ▸ *a* and S4*a*). The FAD-binding loop of FDHs has been hypothesized to adopt two conformations. In apo crystals of Thal the FAD-binding loop adopts an ‘open’ conformation (Fig. 3 ▸ *c*), whereas in FAD-bound structures the loop closes (Fig. 3 ▸ *d*; Moritzer & Niemann, 2019 ▸). The side chain of a glutamate flanking the FAD-binding loop flips by almost 180° upon FAD binding in Thal (Figs. 3 ▸ *c* and 3 ▸ *d*) and PyrH (Figs. 3 ▸ *e* and 3 ▸ *f*). Although the loop of apo Xcc4156 would be expected to resemble that of apo Thal, it has similarities to both the open and closed states (Fig. 3 ▸ *g*). However, Glu57 in Xcc4156 adopts the same conformation as Glu49 in apo Thal and apo PyrH, indicating that the loop is in its open conformation (Figs. 3 ▸ *a*, 3 ▸ *c* and 3 ▸ *e*). While part of the loop differs between chain *A* and *B* in apo Xcc4156 (Fig. 3 ▸ *b*), probably owing to the crystal contact in chain *A*, Glu57 has the same orientation in both chains.

Instead of FAD, the FAD-binding site in both chains of Xcc4156 contains a well defined l-tartrate ion (Fig. 3 ▸ *h* and Supplementary Figs. S2*a*–S2*c*) which does not directly interact with the loop. Tartrate was used as the precipitant in crystallization. Interestingly, the same tartrate-binding site can be observed in two other halogenases: an unnamed halogenase and BrvH (PDB entries 2pyx and 6frl; Joint Center for Structural Genomics, unpublished work; Neubauer *et al.*, 2018 ▸; Supplementary Figs. S2*d* and S2*e*). The coordination is the same in all three cases, with most adjacent water molecules at the same positions and the involvement of three backbone N atoms (Thr23, Ala24 and Leu335 in Xcc4156; Thr16, Ala17 and selenomethionine Met351 in PDB entry 2pyx; and Thr19, Ala20 and Leu340 in BrvH). Two corresponding residues (Ala16 and Leu349) in Thal coordinate one phosphate of the FAD (PDB entry 6sls; Supplementary Fig. S2*f*).

Another striking feature of the FAD-binding site is the side chain of Asn347 (Supplementary Fig. S3). This amino acid is the second amino acid of the canonical halide-binding site that has been observed in FDHs (Supplementary Fig. S5). The latter site is close to the isoalloxazine ring of the FAD and consists of two backbone NH groups that coordinate the halide. Whether this site is relevant for halogenation or is only an artefact of crystallization remains unclear (Blasiak & Drennan, 2009 ▸). In apo Xcc4156, this site (Thr346 and Asn347) does not contain any bound halide. In most FDHs, a glycine occupies the second position of this conserved region. This led Gkotsi *et al.* (2019 ▸) to postulate the halogenase-recognition sequence motif F*x*.P*x*.S*x*.G (where ‘*x*.’ denotes any number of amino acids) in addition to the established G*x*G*xx*G and W*x*W*x*IP motifs. The glycine residue in their proposed sequence motif is conserved in almost all known FDHs. Exceptions are BrvH and the other two *X. campestris* halogenases characterized by the Sewald laboratory (Neubauer *et al.*, 2018 ▸; Ismail *et al.*, 2019 ▸), which all have a serine at this position. The asparagine in Xcc4156 is particularly interesting as its side chain occupies the position that would be taken up by the ribityl moiety of FAD in a FAD-bound halogenase, thus possibly hampering FAD binding (Supplementary Figs. S3*a* and S3*b*). The amide N atom is within 1 Å of the C_2_′ atom of a FAD molecule from Thal when superimposed with Xcc4156.

### 3.2. Soaking of apo Xcc4156 crystals   

**Figure 4:**
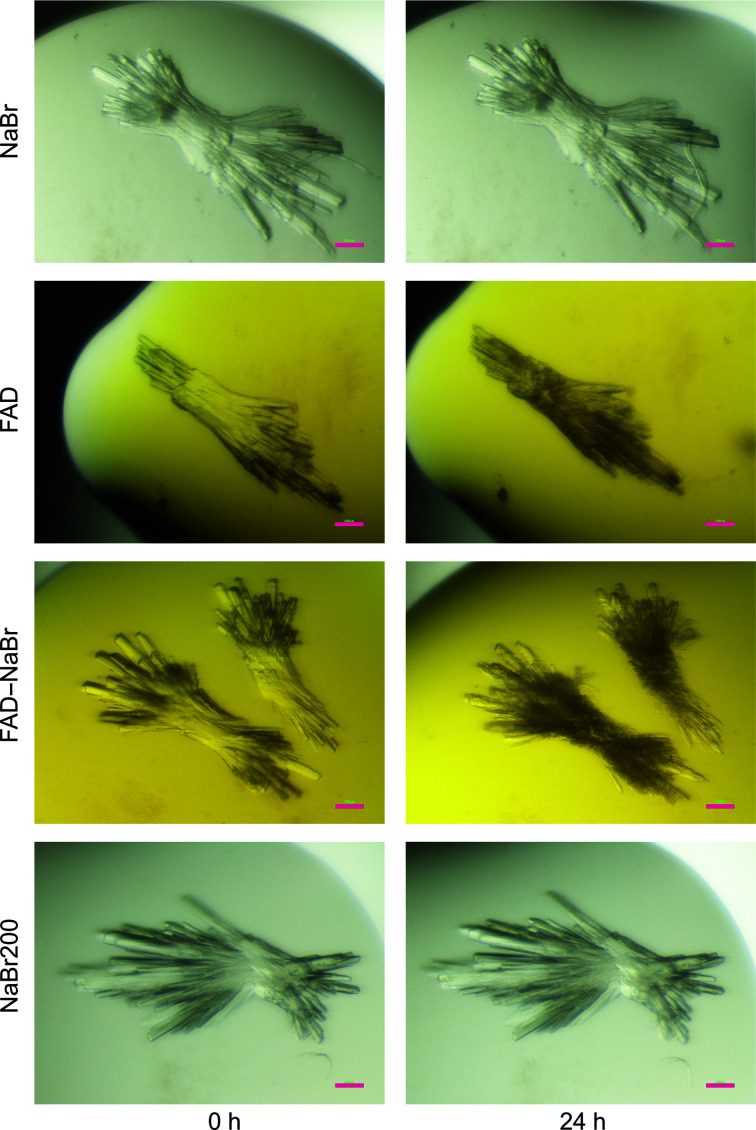
Soaking of apo crystals of Xcc4156. Soaks were performed by the addition of 0.5 µl 150 m*M* NaBr (‘NaBr’), 15 m*M* FAD (’FAD’), 15 m*M* FAD and 150 m*M* NaBr (‘FAD–NaBr’) or 200 m*M* NaBr (‘NaBr200’) in reservoir solution to a drop consisting of 0.5 µl reservoir solution and 1 µl protein solution. Photographs were taken directly after the addition of the ligand solution to the drop (‘0 h’) and after 24 h. The pink bar indicates 100 µm.

After unsuccessfully attempting to cocrystallize Xcc4156 with its cofactor FAD and NaBr (Supplementary Table S2), soaking of apo crystals was performed. Thin, intergrown needles grew within one or a few days of setting up the plates. For soaking, reservoir solution containing the respective ligand(s) was added to the drop (Fig. 4 ▸).

When 150 m*M* NaBr was added, the crystals were not harmed and retained their appearance. The addition of 15 m*M* FAD led to a visual deterioration of the crystals, and the addition of both 15 m*M* FAD and 150 m*M* NaBr led to a worse crumbling of the crystals. To verify that this effect was not caused by the higher ionic strength of the latter solution, crystals were soaked with 200 m*M* NaBr. These crystals, like those at a lower NaBr concentration, did not show any sign of harm. Interestingly, the crumbling of the crystals appeared to be much worse for smaller crystals and intergrown areas, suggesting that single large crystals are less prone to deterioration.

### 3.3. Crystal structures of soaked Xcc4156   

**Figure 5:**
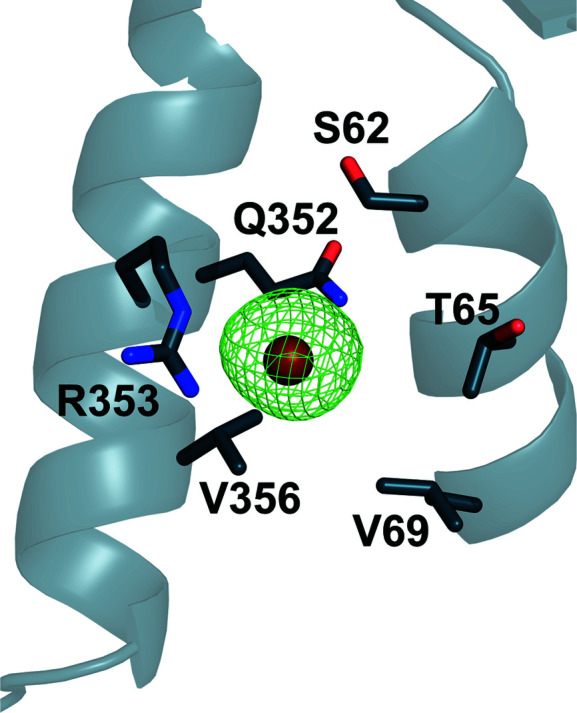
Anomalous difference density of a bromide ion in an Xcc4156 crystal soaked with NaBr. The bromide ion is coloured brown; the anomalous difference density (green mesh) is contoured at 3σ. Side chains at a distance of less than 6 Å from the bromide are shown as sticks.

The soaked crystals were harvested and data were collected (see Table 1 ▸ for data statistics). The structures were solved by refinement of the final apo model against the respective data. For crystals soaked with NaBr no bromide could be observed in the halide-binding site despite the presence of bromide in other sites as confirmed by an anomalous difference map (Fig. 5 ▸).

When data sets were collected from crystals soaked with FAD (or FAD and NaBr), we noticed that around half of the crystals showed little to no diffraction (to less than 4 Å resolution, compared with around 2–3 Å for apo crystals or NaBr soaks). Interestingly, in all of the remaining data sets (one and three data sets from crystals soaked with FAD or with FAD and NaBr, respectively) the FAD-binding sites were empty save for tartrate, which was present at the same site as in the apo crystals in both chains. The crystals soaked with FAD thus showed similar electron density to the apo crystals, whereas those soaked with both FAD and NaBr resembled the NaBr-soaked crystals. As the soaked structures did not reveal any relevant structural information beyond the apo structure and had considerably lower resolution, we did not perform further model building or deposit these structures in the PDB.

The visual deterioration of the crystals, the partial loss of diffraction and the finding that no electron density for FAD was present upon soaking suggest that binding of FAD might destroy the crystals. When no FAD is bound the crystals still diffract, but the resulting electron density shows no sign of FAD, and when FAD is bound the diffraction is gravely reduced.

## 4. Discussion   

We observed a visual deterioration of apo crystals of the flavin-dependent halogenase Xcc4156 upon the addition of FAD, which was even more dramatic when bromide was present as well. This might be explained by the binding of FAD causing a change in the position of the FAD-binding loop. Indeed, the loop is partly disordered in one chain of the apo structure and is fixed in an open conformation in the other chain.

There are four other halogenases, RebH, PyrH, PltM and Thal, for which both apo and holo structures are available. In apo PyrH, the FAD-binding loop (amino acids 37–43) is disordered and was not modelled (Zhu *et al.*, 2009 ▸). In the presence of FAD the loop adopts an ordered ‘closed’ conformation. The loop is close to the FAD and interacts with it through backbone atoms. It should be mentioned that the apo and FAD-bound structures of PyrH have different space groups and crystal packing, the reasons for which are unclear as the crystals grew under the same conditions. Thal behaves similarly to PyrH, with the exception that in the apo form it exhibits a well defined loop in the ‘open’ conformation, leaving space between the loop and the potential FAD. The loop closes when Thal is soaked with FAD (Moritzer & Niemann, 2019 ▸). In both RebH and PltM the FAD-binding loop, as in Thal, is well defined in the apo state; however, the loop position does not change in the presence of the cofactor. This can be explained as in both structures the FAD-binding loop is part of a crystal contact, with the loops of two chains interacting with each other, generating a noncrystallographic twofold axis. In PltM, the conformation is very similar to that observed for ‘closed’ loop conformations (Mori *et al.*, 2019 ▸). This is in contrast to the ‘open’ loop position in RebH (Bitto *et al.*, 2008 ▸), where the loop is not in direct contact with the cofactor, leaving the FAD-binding site solvent exposed. Unlike in RebH and PltM, soaking Xcc4156 crystals with FAD seems to have caused the FAD-binding loop to change its position, resulting in visible harm to the crystals.

In order to explain this different behaviour, the crystal contacts that the loops are involved in were compared using the *PISA* server (Krissinel & Henrick, 2007 ▸). In Xcc4156 only four residues are part of the relevant interface, as opposed to six or five residues in apo RebH and apo PltM, respectively. The average buried area of the residues within the loop is 55% for RebH, 42% or 54% for PltM (with four monomers in the asymmetric unit; thus, two dimers that interact via their loops) and only 27.5% for Xcc4156. This might indicate a weaker interaction that can be disrupted as FAD binds. Binding of FAD in chain *A* and the resulting elimination of the crystal contact seems to have led to a loss in diffraction of the crystals. The crystals of Xcc4156 that still showed diffraction had no FAD bound in chain *A*, as would be expected. Interestingly, however, soaking the crystals with FAD also did not result in FAD being bound by the less ordered FAD-binding loop (chain *B*) of the crystals. The high concentration of tartrate (600 m*M* compared with 15 m*M* FAD) and its presence in the FAD-binding site could explain the absence of FAD but not the observed crystal cracking.

We also observed that the visual deterioration of the crystals during soaking was more severe when NaBr was present in addition to FAD, which could not be explained by the higher ionic strength of the solution. However, the halide binding might have a cooperative effect, promoting the binding of FAD. This would enable the soaking solution to affect the crystals more dramatically. Without FAD, even in the presence of high concentrations of NaBr no halide is bound in the proposed halide-binding site. This is in accordance with the literature. Out of 50 published structures of FDHs (as of 8 January 2020), 15 have no FAD and no halide bound in the respective binding sites (apo), 16 have only FAD modelled (either because no halide is present or because it was not modelled) and another 12 have both FAD and the halide modelled in at least one chain. Of the seven remaining structures, one PrnA structure (PDB entry 2ard) contains FADH_2_ with no halide modelled (Dong *et al.*, 2005 ▸) and two PltM structures (PDB entries 6bza and 6bzz) contain FAD that is bound in an unusual pose (Mori *et al.*, 2019 ▸). One RebH structure (PDB entry 2oa1) contains FAD and chloride in one chain, but only adenine and no halide modelled in the other (Bitto *et al.*, 2008 ▸), and one Thal structure (PDB entry 6slt) contains only FAD in one chain, while in the other only AMP was modelled (Moritzer & Niemann, 2019 ▸). The modelling of only parts of the FAD in the latter two cases can be explained as the adenine moiety is bound by a nonflexible region and the riboflavin moiety (which forms part of the halide-binding site) is more flexible in these cases, making its modelling harder or impossible (Moritzer & Niemann, 2019 ▸). The MibH structure (PDB entry 5uao) contains both FAD and chloride in two chains and neither of them in two other chains (Ortega *et al.*, 2017 ▸). The PltA structure (PDB entry 5dbj) contains FAD in all five chains, but chloride is only present in four chains (Pang *et al.*, 2015 ▸). The correlation between the binding of FAD and the halide strongly implies a dependence of halide binding on FAD binding, as has been proposed by Mori *et al.* (2019 ▸). The cooperative effect that we observed substantiates our hypothesis that FAD binding causes the loop to close and the crystals to break and makes a nonspecific effect of FAD binding unlikely. As bromide soaks on their own do not negatively affect the crystal integrity, it is hard to rationalize the exacerbated damage to crystals upon soaking with FAD and bromide other than through cooperative binding at the evolved halide-binding site formed by the protein and FAD. We cannot completely rule out that nonspecific binding of bromide and FAD to the protein surface causes this effect, but this appears to us to be highly unlikely.

In summary, we observed the cracking of apo crystals of the halogenase Xcc4156 upon the addition of FAD to the crystals. This could be explained by a change in the position of the FAD-binding loop, which was part of a crystal contact. This effect was increased in the presence of bromide, suggesting a cooperative effect between the binding of FAD and the halide.

## Supplementary Material

PDB reference: Xcc4156, 6y1w

Supplementary Figures, SupplementaryTables and list of halogenase sequences. DOI: 10.1107/S2059798320007731/ag5038sup1.pdf

## Figures and Tables

**Table 1:** X-ray data-collection and refinement statistics The data-collection statistics were taken from the *AIMLESS* output; the refinement and model statistics were taken from *phenix.table_one*. For the data sets containing Br, the statistics were calculated treating Friedel mates as different reflections, except for the values marked with an asterisk.

	Apo Xcc4156	Xcc4156 + FAD	Xcc4156 + NaBr	Xcc4156 + FAD/NaBr
PDB code	6y1w	—	—	—
Space group	*P*3_2_	*P*3_2_	*P*3_2_	*P*3_2_
Unit-cell parameters (Å, °)	*a* = *b* = 120.0, *c* = 70.4, α = β = 90, γ = 120	*a* = *b* = 119.9, *c* = 69.7, α = β = 90, γ = 120	*a* = *b* = 120.1, *c* = 70.2, α = β = 90, γ = 120	*a* = *b* = 119.7, *c* = 69.9, α = β = 90, γ = 120
Data-collection statistics				
Wavelength (Å)	0.91714	0.9184	0.9184	0.9184
Resolution range (Å)	45.66–1.60 (1.63–1.60)	45.45–2.40 (2.49–2.40)	45.63–2.00 (2.04–2.00)	45.47–2.30 (2.38–2.30)
No. of measured reflections	1551581 (69795)	462687 (48945)	799702 (45171)	521479 (47365)
No. of unique reflections	149524 (7328)	43802 (4635)	76232 (4384)*	49785 (4577)*
Completeness (%)	100 (100)	100 (100)	99.3 (95.5)	99.9 (99.8)
*R*_merge_ (%)	9.4 (139.6)	12.1 (144.9)	16.2 (145.1)	13.7 (98.5)
*R*_meas_ (%)	9.9 (147.6)	12.7 (152.2)	18.0 (161.1)	15.2 (109.7)
Multiplicity	10.4 (9.5)	10.6 (10.6)	5.2 (5.2)	5.2 (5.2)
Mean *I*/σ(*I*)	17.1 (1.7)	15.8 (1.7)	13.7 (1.6)	14.4 (2.3)
CC_1/2_ (%)	99.9 (60.4)	99.9 (64.5)	99.7 (56.5)	99.8 (74.8)
Refinement and model statistics				
No. of reflections (working/test)	142174/7318	41621/2133	144758/7304	94652/4789
*R*_cryst_ (%)	15.8	18.3	19.1	18.1
*R*_free_ (%)	18.9	23.8	22.8	21.7
R.m.s.d., bonds (Å)	0.011	0.006	0.003	0.004
R.m.s.d., angles (°)	1.11	0.88	0.67	0.75
Average *B* factor (Å^2^)				
Overall	24.4	56.2	28.5	38.3
Protein	22.6	54.9	27.0	37.1
Ligands	33.3	—	—	—
Solvent	35.3	65.4	38.44	46.8
No. of atoms				
Total	9532	9351	9351	9351
Protein	8154	8154	8154	8154
Ligands	181	—	—	—
Solvent	1197	1197	1197	1197
Ramachandran plot from *MolProbity*				
Favoured region (%)	98.29	98.09	98.09	98.09
Allowed region (%)	1.71	1.91	1.91	1.91

**Table 2:** List of halogenases structurally compared with Xcc4156 chain *A* The colours are in accordance with Fig. 3 ▸, grouping the halogenases that belong to the C5 Trp halogenase subgroup (red), the C7 Trp halogenase subgroup (green), the Xcc4156/BrvH subgroup (blue) and an ‘outgroup’ of other halogenases that are less structurally related to Xcc4156 (grey; refer to Supplementary Fig. S1 for a phylogenetic tree). The description gives an idea of the halogenase family that each listed halogenase belongs to. Variant B halogenases act on carrier protein-tethered substrates. The sequence identities output by *Clustal Omega* were obtained from sequence-based alignment. The structural similarity was output by the *DALI* ‘all-against-all structure comparison’ as a similarity matrix. For halogenases where multiple structures have been published or multiple chains were present in the asymmetric unit, the PDB code and chain were used that had the highest similarity to Xcc4156 chain *A*. The ligands present in the structure were mostly the cofactor FAD and halide Cl and, in the case of PrnA, the halogenation product 7-chlorotryptophan. Ligands in italics are presumably irrelevant and are only present owing to the crystallization or cryoprotection conditions. CL, chloride; CTE, 7-chlorotryptophan; EDO, 1,2-ethanediol; FAD, flavin adenine dinucleotide; GOL, glycerol; MES, 2-(*N*-morpholino)-ethanesulfonic acid; PO4, phosphate ion; SO4, sulfate ion; TLA, L-(+)-tartaric acid.

Name	Description	Sequence identity (*Clustal Omega*) (%)	Structural similarity score (*DALI*)	PDB code and chain ID	Ligands in chain	Reference
BrvH	Indole halogenase	45.5	50.4	6frl chain *A*	*TLA*, *MES*, *GOL*	Neubauer *et al.* (2018 ▸)
PyrH	Trp-5 halogenase	33.7	46.8	2wes chain *A*	FAD, CL	Zhu *et al.* (2009 ▸)
SttH	Trp-6 halogenase	34.0	45.7	5hy5 chain *B*	FAD, CL	Shepherd *et al.* (2016 ▸)
Tar14	Trp-6 halogenase	32.0	45.0	6nsd chain *B*	FAD, *SO4*	Luhavaya *et al.* (2019 ▸)
PrnA	Trp-7 halogenase	32.3	44.8	2ar8 chain *A*	FAD, CL, CTE	Dong *et al.* (2005 ▸)
Thal	Trp-6 halogenase	32.6	44.4	6h43 chain *B*	*PO4, GOL*	Moritzer *et al.* (2019 ▸)
Th-Hal	Trp-6 halogenase	33.7	43.9	5lv9 chain *B*	—	Menon *et al.* (2016 ▸)
RebH	Trp-7 halogenase	34.1	43.8	2oal chain *B*	FAD	Yeh *et al.* (2007 ▸)
MibH	Peptide halogenase	26.4	39.9	5uao chain *C*	—	Ortega *et al.* (2017 ▸)
Mpy16	Pyrrole halogenase (variant B)	18.1	28.1	5buk chain *A*	FAD, *GOL*	El Gamal *et al.* (2016 ▸)
CndH	Phenol halogenase (variant B)	16.8	27.4	3e1t chain *A*	FAD, CL	Buedenbender *et al.* (2009 ▸)
Bmp2	Pyrrole halogenase (variant B)	15.3	26.7	5bva chain *A*	FAD, *EDO*	El Gamal *et al.* (2016 ▸)

## References

[bb1] Agarwal, V., El Gamal, A. A., Yamanaka, K., Poth, D., Kersten, R. D., Schorn, M., Allen, E. E. & Moore, B. S. (2014). *Nat. Chem. Biol.* **10**, 640–647. 10.1038/nchembio.1564 PMC4104138 24974229

[bb2] Ainsley, J., Mulholland, A. J., Black, G. W., Sparagano, O., Christov, C. Z. & Karabencheva-Christova, T. G. (2018). *ACS Omega*, **3**, 4847–4859. 10.1021/acsomega.8b00385 PMC6641897 31458701

[bb3] Bitto, E., Huang, Y., Bingman, C. A., Singh, S., Thorson, J. S. & Phillips, G. N. Jr (2008). *Proteins*, **70**, 289–293. 10.1002/prot.21627 17876823

[bb4] Blasiak, L. C. & Drennan, C. L. (2009). *Acc. Chem. Res.* **42**, 147–155. 10.1021/ar800088r PMC3980734 18774824

[bb5] Buedenbender, S., Rachid, S., Müller, R. & Schulz, G. E. (2009). *J. Mol. Biol.* **385**, 520–530. 10.1016/j.jmb.2008.10.057 19000696

[bb6] Chen, V. B., Arendall, W. B., Headd, J. J., Keedy, D. A., Immormino, R. M., Kapral, G. J., Murray, L. W., Richardson, J. S. & Richardson, D. C. (2010). *Acta Cryst.* D**66**, 12–21. 10.1107/S0907444909042073 PMC2803126 20057044

[bb7] Cowtan, K. (2006). *Acta Cryst.* D**62**, 1002–1011. 10.1107/S0907444906022116 16929101

[bb8] Domergue, J., Erdmann, D., Fossey-Jouenne, A., Petit, J.-L., Debard, A., de Berardinis, V., Vergne-Vaxelaire, C. & Zaparucha, A. (2019). *AMB Express*, **9**, 175. 10.1186/s13568-019-0898-y PMC6823310 31673806

[bb9] Dong, C., Flecks, S., Unversucht, S., Haupt, C., van Pée, K.-H. & Naismith, J. H. (2005). *Science*, **309**, 2216–2219. 10.1126/science.1116510 PMC3315827 16195462

[bb10] Dorrestein, P. C., Yeh, E., Garneau-Tsodikova, S., Kelleher, N. L. & Walsh, C. T. (2005). *Proc. Natl Acad. Sci. USA*, **102**, 13843–13848. 10.1073/pnas.0506964102 PMC1236592 16162666

[bb11] Durak, L. J., Payne, J. T. & Lewis, J. C. (2016). *ACS Catal.* **6**, 1451–1454. 10.1021/acscatal.5b02558 PMC4890977 27274902

[bb12] El Gamal, A., Agarwal, V., Diethelm, S., Rahman, I., Schorn, M. A., Sneed, J. M., Louie, G. V., Whalen, K. E., Mincer, T. J., Noel, J. P., Paul, V. J. & Moore, B. S. (2016). *Proc. Natl Acad. Sci. USA*, **113**, 3797–3802. 10.1073/pnas.1519695113 PMC4833250 27001835

[bb13] Emsley, P., Lohkamp, B., Scott, W. G. & Cowtan, K. (2010). *Acta Cryst.* D**66**, 486–501. 10.1107/S0907444910007493 PMC2852313 20383002

[bb14] Evans, P. R. & Murshudov, G. N. (2013). *Acta Cryst.* D**69**, 1204–1214. 10.1107/S0907444913000061 PMC3689523 23793146

[bb15] Fisher, B. F., Snodgrass, H. M., Jones, K. A., Andorfer, M. C. & Lewis, J. C. (2019). *ACS Cent. Sci.* **5**, 1844–1856. 10.1021/acscentsci.9b00835 PMC6891866 31807686

[bb16] Flecks, S., Patallo, E. P., Zhu, X., Ernyei, A. J., Seifert, G., Schneider, A., Dong, C., Naismith, J. H. & van Pée, K.-H. (2008). *Angew. Chem. Int. Ed.* **47**, 9533–9536. 10.1002/anie.200802466 PMC3326536 18979475

[bb17] Fraley, A. E., Garcia-Borràs, M., Tripathi, A., Khare, D., Mercado-Marin, E. V., Tran, H., Dan, Q., Webb, G. P., Watts, K. R., Crews, P., Sarpong, R., Williams, R. M., Smith, J. L., Houk, K. N. & Sherman, D. H. (2017). *J. Am. Chem. Soc.* **139**, 12060–12068. 10.1021/jacs.7b06773 PMC5595095 28777910

[bb18] Frese, M., Schnepel, C., Minges, H., Voss, H., Feiner, R. & Sewald, N. (2016). *ChemCatChem*, **8**, 1799–1803.

[bb19] Gerlach, M., Mueller, U. & Weiss, M. S. (2016). *J. Large-Scale Res. Facil.* **2**, A47.

[bb20] Gkotsi, D. S., Ludewig, H., Sharma, S. V., Connolly, J. A., Dhaliwal, J., Wang, Y., Unsworth, W. P., Taylor, R. J. K., McLachlan, M. M. W., Shanahan, S., Naismith, J. H. & Goss, R. J. M. (2019). *Nat. Chem.* **11**, 1091–1097. 10.1038/s41557-019-0349-z PMC6875430 31611633

[bb21] Hammer, P. E., Hill, D. S., Lam, S. T., Van Pée, K.-H. & Ligon, J. M. (1997). *Appl. Environ. Microbiol.* **63**, 2147–2154. 10.1128/aem.63.6.2147-2154.1997 PMC168505 9172332

[bb22] Heemstra, J. R. & Walsh, C. T. (2008). *J. Am. Chem. Soc.* **130**, 14024–14025. 10.1021/ja806467a PMC2726796 18828589

[bb23] Holm, L. (2019). *Bioinformatics*, **35**, 5326–5327. 10.1093/bioinformatics/btz536 31263867

[bb24] Ismail, M., Frese, M., Patschkowski, T., Ortseifen, V., Niehaus, K. & Sewald, N. (2019). *Adv. Synth. Catal.* **361**, 2475–2486.

[bb25] Kabsch, W. (2010). *Acta Cryst.* D**66**, 125–132. 10.1107/S0907444909047337 PMC2815665 20124692

[bb26] Keegan, R. M. & Winn, M. D. (2007). *Acta Cryst.* D**63**, 447–457. 10.1107/S0907444907002661 17372348

[bb27] Keller, S., Wage, T., Hohaus, K., Hölzer, M., Eichhorn, E. & van Pée, K.-H. (2000). *Angew. Chem. Int. Ed.* **39**, 2300–2302. 10.1002/1521-3773(20000703)39:13<2300::aid-anie2300>3.0.co;2-i 10941070

[bb28] Krissinel, E. & Henrick, K. (2007). *J. Mol. Biol.* **372**, 774–797. 10.1016/j.jmb.2007.05.022 17681537

[bb29] Liebschner, D., Afonine, P. V., Baker, M. L., Bunkóczi, G., Chen, V. B., Croll, T. I., Hintze, B., Hung, L.-W., Jain, S., McCoy, A. J., Moriarty, N. W., Oeffner, R. D., Poon, B. K., Prisant, M. G., Read, R. J., Richardson, J. S., Richardson, D. C., Sammito, M. D., Sobolev, O. V., Stockwell, D. H., Terwilliger, T. C., Urzhumtsev, A. G., Videau, L. L., Williams, C. J. & Adams, P. D. (2019). *Acta Cryst.* D**75**, 861–877. 10.1107/S2059798319011471 PMC6778852 31588918

[bb30] Lingkon, K. & Bellizzi, J. J. (2020). *ChemBioChem*, **21**, 1121–1128. 10.1002/cbic.201900667 31692209

[bb31] Livingstone, C. D. & Barton, G. J. (1993). *Comput. Appl. Biosci.* **9**, 745–756. 10.1093/bioinformatics/9.6.745 8143162

[bb32] Luhavaya, H., Sigrist, R., Chekan, J. R., McKinnie, S. M. K. & Moore, B. S. (2019). *Angew. Chem. Int. Ed.* **58**, 8394–8399. 10.1002/anie.201901571 PMC6555645 30963655

[bb33] Ma, L., Zhang, W., Zhu, Y., Zhang, G., Zhang, H., Zhang, Q., Zhang, L., Yuan, C. & Zhang, C. (2017). *Appl. Biochem. Biotechnol.* **101**, 6123–6136. 10.1007/s00253-017-8375-5 28620687

[bb34] Madeira, F., Park, Y. M., Lee, J., Buso, N., Gur, T., Madhusoodanan, N., Basutkar, P., Tivey, A. R. N., Potter, S. C., Finn, R. D. & Lopez, R. (2019). *Nucleic Acids Res.* **47**, W636–W641. 10.1093/nar/gkz268 PMC6602479 30976793

[bb35] McCoy, A. J., Grosse-Kunstleve, R. W., Adams, P. D., Winn, M. D., Storoni, L. C. & Read, R. J. (2007). *J. Appl. Cryst.* **40**, 658–674. 10.1107/S0021889807021206 PMC2483472 19461840

[bb36] Menon, B. R. K., Brandenburger, E., Sharif, H. H., Klemstein, U., Shepherd, S. A., Greaney, M. F. & Micklefield, J. (2017). *Angew. Chem. Int. Ed.* **56**, 11841–11845. 10.1002/anie.201706342 PMC5637929 28722773

[bb37] Menon, B. R. K., Latham, J., Dunstan, M. S., Brandenburger, E., Klemstein, U., Leys, D., Karthikeyan, C., Greaney, M. F., Shepherd, S. A. & Micklefield, J. (2016). *Org. Biomol. Chem.* **14**, 9354–9361. 10.1039/c6ob01861k 27714222

[bb38] Minges, H., Schnepel, C., Böttcher, D., Weiss, M. S., Spross, J., Bornscheuer, U. T. & Sewald, N. (2020). *ChemCatChem*, **12**, 818–831.

[bb39] Mori, S., Pang, A. H., Thamban Chandrika, N., Garneau-Tsodikova, S. & Tsodikov, O. V. (2019). *Nat. Commun.* **10**, 1255. 10.1038/s41467-019-09215-9 PMC6424973 30890712

[bb40] Moritzer, A.-C., Minges, H., Prior, T., Frese, M., Sewald, N. & Niemann, H. H. (2019). *J. Biol. Chem.* **294**, 2529–2542. 10.1074/jbc.RA118.005393 PMC6378962 30559288

[bb41] Moritzer, A.-C. & Niemann, H. H. (2019). *Protein Sci.* **28**, 2112–2118. 10.1002/pro.3739 PMC6863734 31589794

[bb42] Murshudov, G. N., Skubák, P., Lebedev, A. A., Pannu, N. S., Steiner, R. A., Nicholls, R. A., Winn, M. D., Long, F. & Vagin, A. A. (2011). *Acta Cryst.* D**67**, 355–367. 10.1107/S0907444911001314 PMC3069751 21460454

[bb43] Neubauer, P. R., Widmann, C., Wibberg, D., Schröder, L., Frese, M., Kottke, T., Kalinowski, J., Niemann, H. H. & Sewald, N. (2018). *PLoS One*, **13**, e0196797. 10.1371/journal.pone.0196797 PMC5945002 29746521

[bb44] Neumann, C. S., Walsh, C. T. & Kay, R. R. (2010). *Proc. Natl Acad. Sci. USA*, **107**, 5798–5803. 10.1073/pnas.1001681107 PMC2851905 20231486

[bb45] Ortega, M. A., Cogan, D. P., Mukherjee, S., Garg, N., Li, B., Thibodeaux, G. N., Maffioli, S. I., Donadio, S., Sosio, M., Escano, J., Smith, L., Nair, S. K. & van der Donk, W. A. (2017). *ACS Chem. Biol.* **12**, 548–557. 10.1021/acschembio.6b01031 PMC5315687 28032983

[bb46] Pang, A. H., Garneau-Tsodikova, S. & Tsodikov, O. V. (2015). *J. Struct. Biol.* **192**, 349–357. 10.1016/j.jsb.2015.09.013 26416533

[bb47] Potterton, L., Agirre, J., Ballard, C., Cowtan, K., Dodson, E., Evans, P. R., Jenkins, H. T., Keegan, R., Krissinel, E., Stevenson, K., Lebedev, A., McNicholas, S. J., Nicholls, R. A., Noble, M., Pannu, N. S., Roth, C., Sheldrick, G., Skubak, P., Turkenburg, J., Uski, V., von Delft, F., Waterman, D., Wilson, K., Winn, M. & Wojdyr, M. (2018). *Acta Cryst.* D**74**, 68–84. 10.1107/S2059798317016035 PMC5947771 29533233

[bb48] Robert, X. & Gouet, P. (2014). *Nucleic Acids Res.* **42**, W320–W324. 10.1093/nar/gku316 PMC4086106 24753421

[bb49] Runguphan, W. & O’Connor, S. E. (2013). *Org. Lett.* **15**, 2850–2853. 10.1021/ol401179k 23713451

[bb50] Seibold, C., Schnerr, H., Rumpf, J., Kunzendorf, A., Hatscher, C., Wage, T., Ernyei, A. J., Dong, C., Naismith, J. H. & van Pée, K.-H. (2006). *Biocatal. Biotransformation*, **24**, 401–408.

[bb51] Shepherd, S. A., Menon, B. R. K., Fisk, H., Struck, A. W., Levy, C., Leys, D. & Micklefield, J. (2016). *ChemBioChem*, **17**, 821–824. 10.1002/cbic.201600051 PMC5071727 26840773

[bb52] Thapa, H. R., Lail, A. J., Garg, N. & Agarwal, V. (2018). *Methods Enzymol.* **604**, 333–366. 10.1016/bs.mie.2018.01.028 29779658

[bb53] Waterhouse, A. M., Procter, J. B., Martin, D. M. A., Clamp, M. & Barton, G. J. (2009). *Bioinformatics*, **25**, 1189–1191. 10.1093/bioinformatics/btp033 PMC2672624 19151095

[bb54] Yamanaka, K., Ryan, K. S., Gulder, T. A. M., Hughes, C. C. & Moore, B. S. (2012). *J. Am. Chem. Soc.* **134**, 12434–12437. 10.1021/ja305670f PMC3415713 22800473

[bb55] Yeh, E., Blasiak, L. C., Koglin, A., Drennan, C. L. & Walsh, C. T. (2007). *Biochemistry*, **46**, 1284–1292. 10.1021/bi0621213 17260957

[bb56] Yeh, E., Cole, L. J., Barr, E. W., Bollinger, J. M., Ballou, D. P. & Walsh, C. T. (2006). *Biochemistry*, **45**, 7904–7912. 10.1021/bi060607d 16784243

[bb57] Yeh, E., Garneau, S. & Walsh, C. T. (2005). *Proc. Natl Acad. Sci. USA*, **102**, 3960–3965. 10.1073/pnas.0500755102 PMC554827 15743914

[bb58] Zeng, J., Lytle, A. K., Gage, D., Johnson, S. J. & Zhan, J. (2013). *Bioorg. Med. Chem. Lett.* **23**, 1001–1003. 10.1016/j.bmcl.2012.12.038 PMC3557752 23312946

[bb59] Zeng, J. & Zhan, J. (2011). *Biotechnol. Lett.* **33**, 1607–1613. 10.1007/s10529-011-0595-7 21424165

[bb60] Zhu, X., De Laurentis, W., Leang, K., Herrmann, J., Ihlefeld, K., van Pée, K.-H. & Naismith, J. H. (2009). *J. Mol. Biol.* **391**, 74–85. 10.1016/j.jmb.2009.06.008 PMC2713781 19501593

