# Supplementary material for: Structure of apo flavin-dependent halogenase Xcc4156 hints at a reason for cofactor-soaking difficulties
Source: Acta Crystallogr D Struct Biol. 2020 Jun 30;76(Pt 7):687–97. doi: 10.1107/S2059798320007731 (PMC7336383; doi:10.1107/S2059798320007731)
Supplement: Supplementary file 1 [file d-76-00687-sup1.pdf]

Acta Cryst D

STRUCTURAL BIOLOGY

Volume 76 (2020)

Supporting information for article:

Structure of apo flavin-dependent halogenase Xcc4156 hints at a reason for cofactor-soaking difficulties

Christiane Widmann, Mohamed Ismail, Norbert Sewald and Hartmut H. Niemann

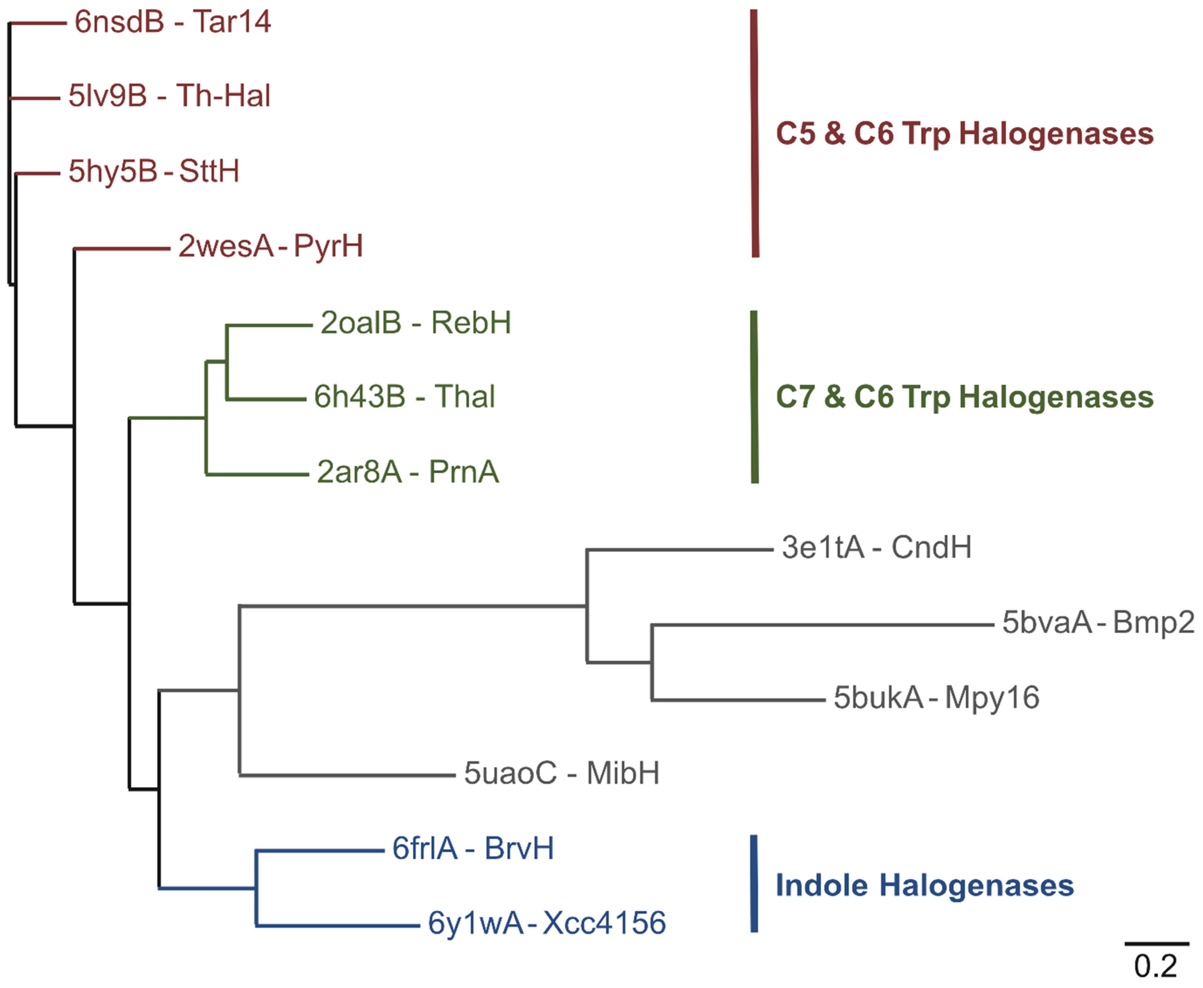

Figure S1 Phylogenetic tree based on the structure comparison output by DALI. The PDB and chain IDs of the respective halogenases are given in addition to their names. The colors are in accordance with Fig. 2, grouping the halogenases that belong to the C5 Trp halogenase subgroup (red), C7 Trp halogenase subgroup (green), Xcc4156/BrvH (blue) and an 'outgroup' of other halogenases that are less structurally related to Xcc4156 (grey; refer to Table 2 for more information). The structure comparison was performed in DALI and the resulting file uploaded to ClustalW2 (Madeira *et al.*, 2019) in order to generate a phylogenetic tree using a neighbor-joining algorithm and distance correction including gaps. The scale bar (bottom right) shows the distance value which is the number of amino acid substitutions as a proportion of the alignment length.

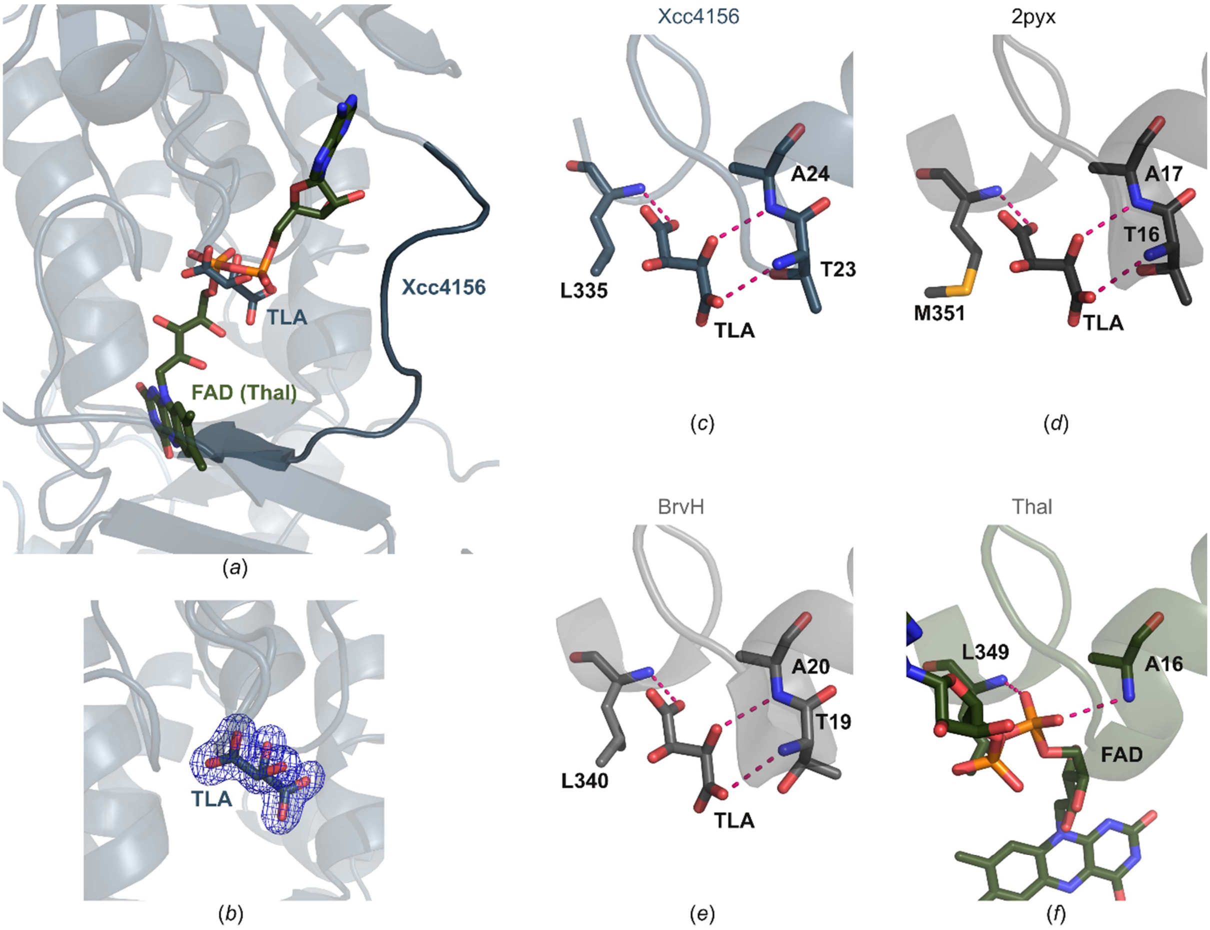

Figure S2 Detailed view of the FAD binding site of Xcc4156. (*a*) The Xcc4156 crystals contain a L-tartrate ion (blue; 'TLA') in the FAD binding site in both chains. Chain *A* of the Xcc4156 structure was superimposed with a Thal structure and the FAD from Thal is shown as sticks (green; PDB entry 6sls chain *A*). The tartrate of Xcc4156 sits in the space that in Thal and other FDHs is occupied by the phosphate groups of FAD. (*b*) Electron density (2Fo-Fc contoured at 1 σ) of the tartrate. (*c*) – (*e*) TLA in Xcc4156 (blue), an unnamed halogenase (black; PDB entry 2pyx chain *A*) and BrvH (grey; PDB entry 6frl chain *A*). In each case, the main chain nitrogen atoms of three surrounding residues (T23, A24 & L335; T16, A17 & M351 and T19, A20 & L340 in Xcc4156, 2pyx and BrvH, respectively) coordinate the tartrate in the same way (pink dotted lines). (*f*) One phosphate of the FAD from Thal (green; PDB entry 6sls chain *A*) is coordinated in a similar way by two main chain nitrogen atoms (of A16 & L349).

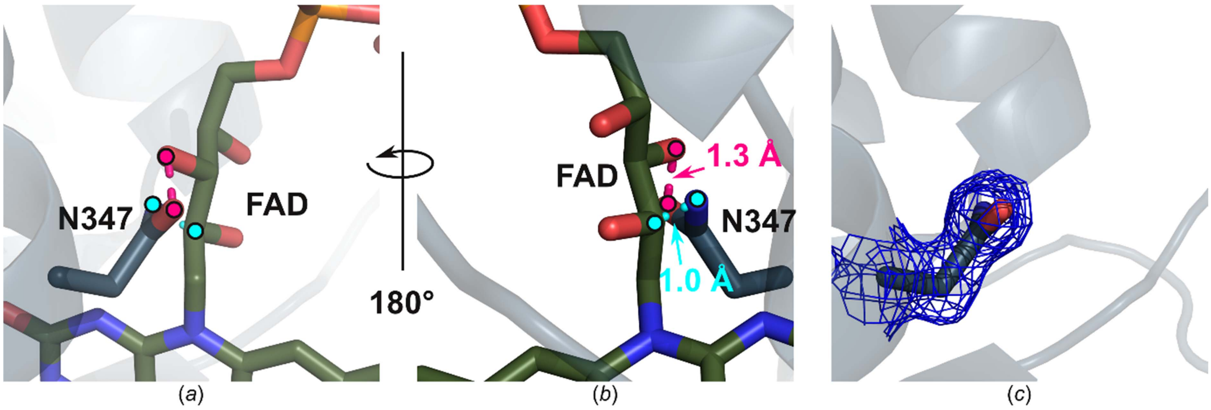

Figure S3 Detailed view of the clash of the N347 position of Xcc4156 chain *A* with FAD from Thal. (*a*) FAD binding site of Xcc4156 (blue) with N347 (part of the halide binding site) and FAD from a Thal structure that was superimposed (green; PDB entry 6sls chain *A*) in order to show the presumed FAD position upon binding. The pink dotted lines connect a ribityl oxygen atom of the FAD with the amide oxygen of the N347 side chain that are only 1.3 Å apart. The cyan dotted lines mark the distance between the amide nitrogen of N347 and a ribityl carbon in FAD that are 1.0 Å apart. (b) The same site, the view is rotated by 180°. Given the proximity of N347 to the presumed FAD position, FAD binding might be sterically hindered in Xcc4156. (*c*) Electron density of the N347 side chain (2Fo-Fc contoured at 1 σ).

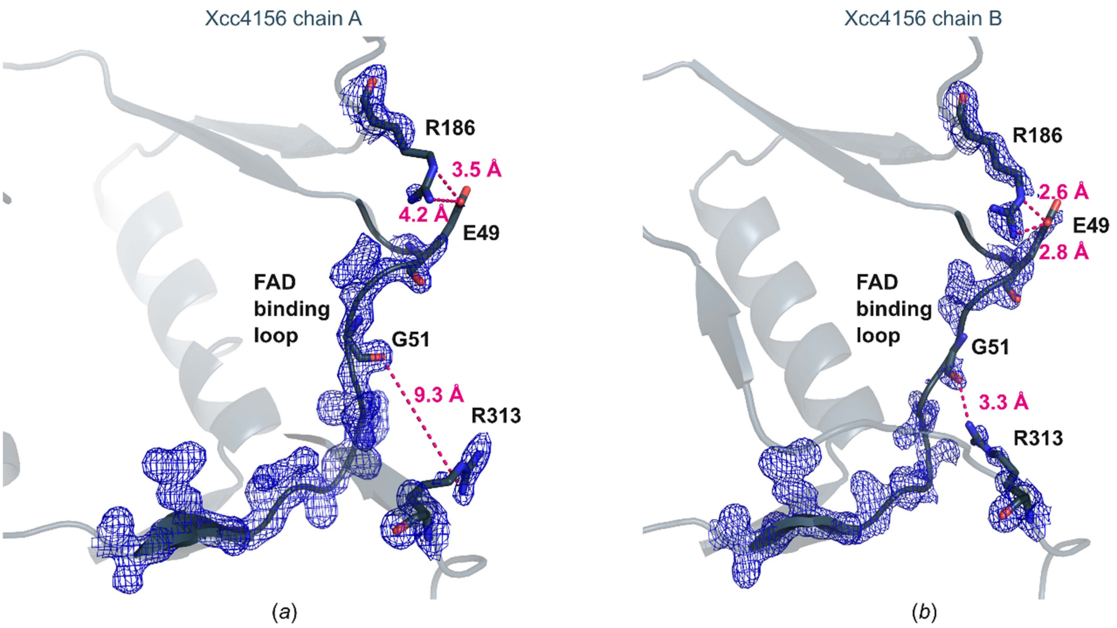

Figure S4 FAD binding loops in apo Xcc4156. (*a*) In chain *A*, the FAD binding loop has continuous and well-defined electron density (2Fo-Fc contoured at 1 σ). (*b*) The electron density in chain *B* is less reliable, indicating a more flexible loop position. Unlike apo PyrH, the FAD binding loop of which was not modelled (Fig. 3*e*), a plausible loop position could be built into the electron density in chain *B* of the apo Xcc4156 structure, suggesting a partial coordination by the surrounding part of the protein. (*a*) & (*b*) In addition to the cartoon representation, amino acids that might coordinate the loop in chain *B* are shown. The ε nitrogen as well as one terminal nitrogen of the guanidino group of R186 are near a carboxyl oxygen of E49 (pink dotted lines; 3.5 Å & 4.2 Å in chain *A*; 2.6 Å and 2.8 Å in chain *B*). This 'upper' part of the loop superimposes in most halogenases, thus the coordination at that position probably does not explain the fact that the rest of the loop has some electron density. Whereas in chain *A*, V53 (not shown; see Fig. 3*a*) is most probably responsible for the well-defined electron density, this role might be played by G51 of chain *B*, the carbonyl oxygen of which is 3.3 Å from the guanidino group of R313 in the modelled conformation. In chain *A*, the side chain of R313 is oriented very differently, resulting in 9.3 Å between the respective atoms.

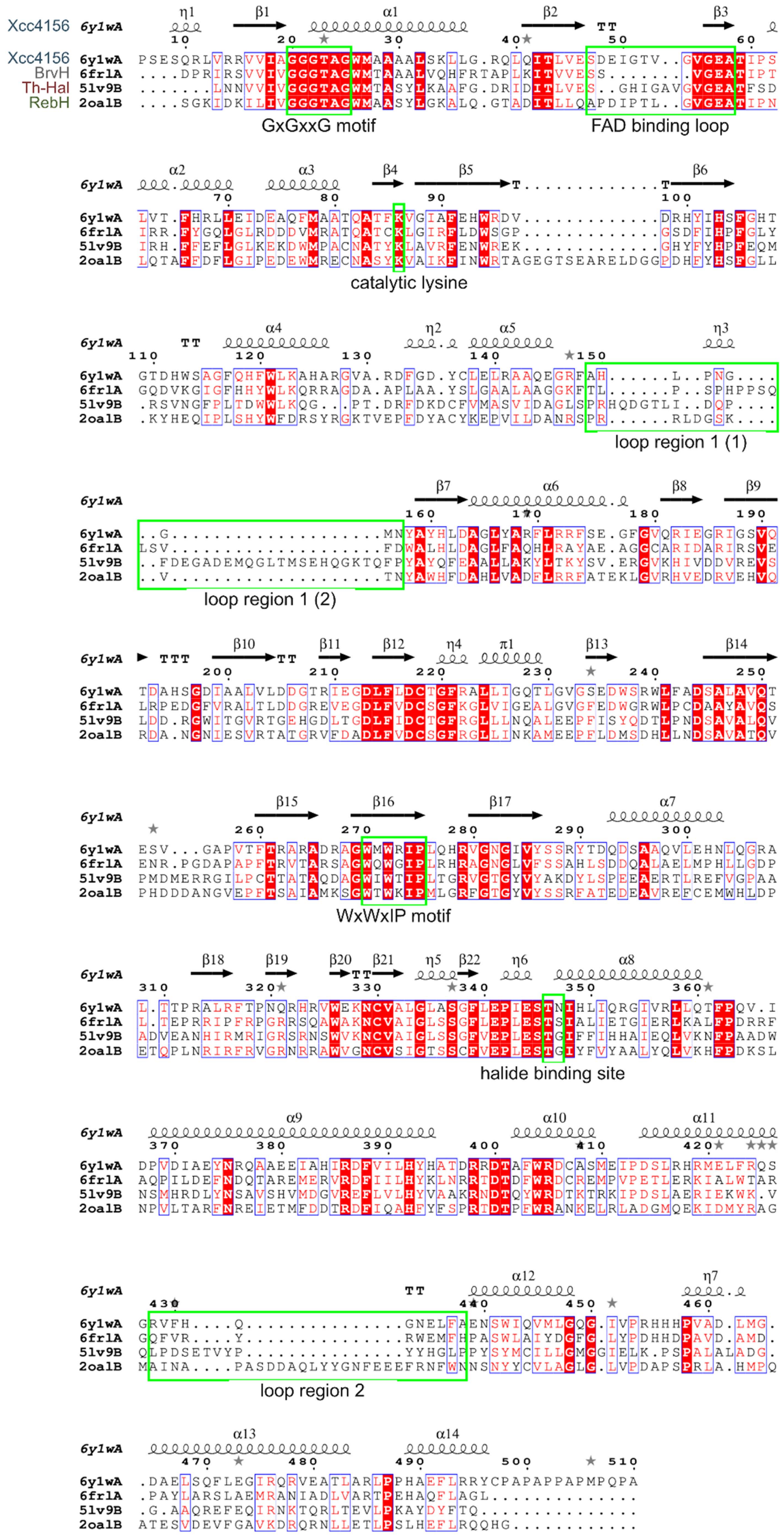
Xcc4156 6y1wA
BrvH 6frlA
Th-Hal 5lv9B
RebH 2oalB
GxGxxG motif
FAD binding loop
catalytic lysine
loop region 1 (1)
loop region 1 (2)
WxWxIP motif
halide binding site
loop region 2

Figure S5 ESPript rendering of the DALI structure comparison of Xcc4156 (PDB entry 6y1w chain *A*), BrvH (PDB entry 6frl chain *A*), Th-Hal (PDB entry 5lv9 chain *B*) and RebH (PDB entry 2oal chain *B*). A red background means that the amino acids in this column (which superimpose in the DALI comparison) are the same; and blue frames around a column mean more than 70 % of its residues are similar according to physico-chemical properties (the respective residues are in red color). Regions mentioned in this paper (GxGxxG motif, FAD binding loop, catalytic lysine, loop region 1, WxWxIP motif, halide binding site & loop region 2) have green frames. The secondary structure and sequence numbering of chain *A* of Xcc4156 are shown above the sequences.

Table S1 Amino acid residues of the catalytic, π stacking and backbone binding regions. The classification according to Moritzer, *et al*. (2019) was used. The amino acids result from the DALI comparison of the protein structures. The colors are in accordance with Fig. 2, grouping the halogenases that belong to the C7 Trp halogenase subgroup (green), C5 Trp halogenase subgroup (red) and the Xcc4156/BrvH subgroup (blue). Italic letters are used to indicate amino acids that are different from the amino acid present in Thal which is the basis of this classification.

<table>
<tr><th>Region</th><th>Thal (6h44)</th><th>RebH (2e4g)</th><th>PrnA (2aqj)</th><th>PyrH (2wet)</th><th>Tar14 (6nsd)</th><th>SttH (5hy5)</th><th>Th-Hal (5lv9)</th><th>BrvH (6frl)</th><th>Xc4156 (6y1w)</th></tr>
<tr><td>Catalytic</td><td>V52</td><td>V52</td><td>I52</td><td>F49</td><td>F62</td><td>F53</td><td>F49</td><td>I57</td><td>I60</td></tr>
<tr><td>Catalytic</td><td>K79</td><td>K79</td><td>K79</td><td>K75</td><td>K88</td><td>K79</td><td>K75</td><td>K83</td><td>K86</td></tr>
<tr><td>Catalytic</td><td>V82</td><td>I82</td><td>I82</td><td>I78</td><td>V91</td><td>V82</td><td>V78</td><td>I86</td><td>I89</td></tr>
<tr><td>Catalytic</td><td>E358</td><td>E357</td><td>E346</td><td>E354</td><td>E373</td><td>E363</td><td>E359</td><td>E349</td><td>E344</td></tr>
<tr><td>Catalytic</td><td>S360</td><td>T359</td><td>T348</td><td>T356</td><td>T375</td><td>T365</td><td>T361</td><td>T351</td><td>T346</td></tr>
<tr><td>π stacking</td><td>P53</td><td>P53</td><td>P53</td><td>S50</td><td>S63</td><td>S54</td><td>S50</td><td>P58</td><td>P61</td></tr>
<tr><td>π stacking</td><td>H110</td><td>H109</td><td>H101</td><td>H92</td><td>H105</td><td>H96</td><td>H92</td><td>H100</td><td>H103</td></tr>
<tr><td>π stacking</td><td>P111</td><td>S110</td><td>L102</td><td>P93</td><td>P106</td><td>P97</td><td>P93</td><td>P101</td><td>S104</td></tr>
<tr><td>π stacking</td><td>F112</td><td>F111</td><td>F103</td><td>F94</td><td>F107</td><td>F98</td><td>F94</td><td>F102</td><td>F105</td></tr>
<tr><td>π stacking</td><td>F465</td><td>F465</td><td>F454</td><td>G450</td><td>G470</td><td>G459</td><td>G455</td><td>M442</td><td>L463</td></tr>
<tr><td>π stacking</td><td>W466</td><td>W466</td><td>W455</td><td>F451</td><td>L471</td><td>L460</td><td>L456</td><td>F443</td><td>F437</td></tr>
<tr><td>Backbone binding</td><td>Y454</td><td>Y454</td><td>Y443</td><td rowspan="3" colspan="4">different substrate-binding loop</td><td rowspan="3" colspan="2">missing substrate-binding loop</td></tr>
<tr><td>Backbone binding</td><td>Y455</td><td>Y455</td><td>Y444</td></tr>
<tr><td>Backbone binding</td><td>E461</td><td>E461</td><td>E450</td></tr>
</table>

Table S2 Screens set up for crystallization of FAD-bound Xcc4156. The drop size was 0.1 µL : 0.1 µL and 0.2 µL : 0.1 µL (protein & ligand : reservoir) in the first nine screening plates – the protein solution thus contained the ligands – , and 0.1 µL : 0.1 µL : 0.1 µL and 0.2 µL : 0.1 µL : 0.1 µL (protein : reservoir : ligand) in the remaining five plates – protein and ligand solution were thus present separately. The given concentrations are those present in the respective solution(s) before mixing with the reservoir solution. The screens were set up twice and the plates were incubated at 4 °C as well as 20 °C.

Screen	Manufacturer	Protein concentration	FAD concentration	NaBr concentration	Drop ratio
Core IV	Qiagen	10 mg /mL	2 mM	50 mM	1:1 & 2:1
Morpheus	Molecular Dimensions	10 mg /mL	2 mM	50 mM	1:1 & 2:1
Core IV	Qiagen	15 mg /mL	3 mM	75 mM	1:1 & 2:1
Morpheus	Molecular Dimensions	15 mg /mL	3 mM	75 mM	1:1 & 2:1
Core IV	Qiagen	7 mg/mL	5 mM	100 mM	1:1 & 2:1
Core IV	Qiagen	5 mg/mL	5 mM	100 mM	1:1 & 2:1
Morpheus	Molecular Dimensions	5 mg/mL	5 mM	100 mM	1:1 & 2:1
Core IV	Qiagen	3 mg/mL	5 mM	100 mM	1:1 & 2:1
Morpheus	Molecular Dimensions	3 mg/mL	5 mM	100 mM	1:1 & 2:1
Midas	Molecular Dimensions	10 mg/mL	5 mM	30 mM	1:1:1 & 2:1:1
Mb class I	Qiagen	10 mg/mL	5 mM	30 mM	1:1:1 & 2:1:1
Mb class II	Qiagen	10 mg/mL	5 mM	30 mM	1:1:1 & 2:1:1
Precipitant Synergy (diluted to 66 %)	Molecular Dimensions	10 mg/mL	5 mM	30 mM	1:1:1 & 2:1:1
Classics Lite Suite	Qiagen	10 mg/mL	5 mM	30 mM	1:1:1 & 2:1:1

S1. List of halogenase sequences for Alignment in Clustal Omega

>AclH
MSIPNRCTVLVVGGGPAGSYAAAALAREGVDTVLLEADVFPRYHIGESMLPSIRHFLRFIDLD
SKFDSYGFVNKNGAAFKLNSKPEAYTDFIAAGGPGSHAWNVVRSEADHLMFKHAGENGAQ
VFDGVKVNSIEFEQIDGLTVDPSLSELGRPVSATWSCKATGGKGSITFEYLIDATGRAGLVST
KYMKNRRYNQGLKNVASWGYWSNAGSYGVGTPREGDPYFEAIEDGSGWVWLIPLHNGTTS
IGVVMNQEAATAKKRETGATTKDLYLNTIKNTPGVWQLLDKAELQSDLKSASDWSYNASSY
ASPYLRIVGDAGCFIDPFFSSGVHLALASGLSAALTIRAAQRGDCDEQAAVSWHSKKVAEGY
TRFLLVVMSALKQISDREKPVLTDFDEDSFNRAFDFFRPIIQGTADVDKNLTQAEIAQTIEFCV
QAFQTASTDEQDAVMTKVAAINSQNGTEGALRELHASLSADERRTLTTIQARQIIRSEDQMNI
DNFTIDVIDGMVPRLERSSLGLARFVPKAQTSREDGLRATLGLPEKQKSIFSY
>AcOTAhal
MAIPQKATVLVIGGGPGGSYSASALAREGIDTVVLEADVFPRYHIGESLVASIRPFLKFIDLDD
TFVNYGFVRKNGAAFKLNNQKEAYTDFILEAGADTFAWNVVRSESDDLMFKHAAKSGAQT
FDGVRVTSIEFTDDDDDDDNNTNRPVSASWKAKDGRTGSIEFDYLVDASGRAGITSTKYLKN
RTFNNYLKNVASWGYWEGATPYGMGTPVEGQPFFEALQDGSGWVWFIPLHNNTTSIGIVMN
QELSTQKKKLSTTTSSRAFYLESLAGARGISRLLDPTTATLTSDIKHASDWSYNASAYGSPYL
RIVGDAGAFIDPYFSSGVHLAVSGGLSAAVSIAASIRGDCPEEAAWKWHSQGVANRYGRFLL
VVLGATKQIRAGDRPVLNGVGDEGFDEAFMVIRPGEFPFFFFSFSFFFLSSEGVGVVVWNCAN
>AoiQ
MTNVPEKCQVLVIGGGPAGSYSASALAREGIDVVLLEAEKFPRYHIGESMLPSMRHFLKFIDA
YDKWDAHGFNVKNGGAFRLNWSRPETYTDFIAAGGPGGYAWNVIRSEADELLFKHAAECG
VKTFDDTKVASIEFAPSEDANPLGRPVSATWTRKDGTSGTLAMDYIVDASGRNGLISTKYLK
NRTFNKGLRNVASWGYWKGGGVHGVGTHKEGAPYFEALKDASGWVWFIPLHNGTHSVGV
VQNQEMATEKKRKMAEPSSKGFYLESLEFVPGIKDLLSNAELISEVKSASDWSYSASNYAFP
GVRIAGDAGSFIDPFFSSGVHLALSGGLSAATTIAAAIRGDCDETVAASWHDKKTSESYTRFL
LVVSSALKQIRSQDEPVISDFDEQSFERAFDLFRPIIQGQADADAKGKLTQAEMSKTVEFCFRA
FAHVSFEEKEALVQKLKSLGHDADANDEANRKALDELEKHLTPEEQAILKTLKGRRMVRPE
DSLNIDNFTLDSIDGLAPRLERGNLGLSPAKKAEVKYTTHDALSFLNGEARAAKKTLSNGESQ
TNGNHLCKDHDQTNGHTAINGHIENNSQGDTNGHTEANGHSQTNGHSNGHAHTNGSHTTN
AHDELNGHNNVEVRSVKSCMADLIAAEKISSQTSIEEGTRHRLISSLHQSAEDLETPFDTVVRL
VDAGRQTAMVCIGGDLGIFKSLVESKRPLSAEELAKATMADPLLVARIMRYMVASRLVGET
GPDQYVASKKTYVFADPRIEHPIRFFHAFSNPAFHALPEFLKETGYQNEPKGSAFQKALNTDL
EPYPWLKQHPDVLKNFQAAMRLTRDANGVDMMPLDQSVSIGHDGAMFVDIGGNTGHQAA
EVLSKYPELAGRVIVQDRGEVIKCAPDIKGIQWMEHDFFQTQPVKGAKYYYLRAILHNWDD

KNTVQILSNIVPAMSADSLVAIDEVVVPEENAHVWPAGLDLQMYSLFSTTERTASQWDAILD
KAGLRAVAVKKYAPVMQSSVIFAAAK
>ArmH1
MEEQVPSSANILVIGGGPAGSYAATVLAREGFEVILLEKDVFPRYHIGESMLPSCRPFLKFIDC
EEKLKNHGFTMKPGAAVKLNQYKREGYTDFISTNPDNAARNVVRSEFDELLLRHASEMGVH
VYEGVRVEEIHFAPDEPTRPISLTWSKEDKTRGTVSFNWLVDASGRNGLMSTRYLKNRTFNK
TLRNVAVWGYWTGTSCYAPGTTRENAPWFEALIDETGWAWFIPLHNGTTSVGVVLVEEESK
RKKSQYRSECKDKSPSEIQHDCYMADLQRAPGLIQLLGTATFEGKLMSAGDYSYHATEYAG
SNFRLAGDAGAFIDPFFSSGIHLALTGGLSAASTIAASIRGDCTESEACGFHNSKVGTAYTRFL
LVVLGVYKQIRSQETAVLYDVDEDNFDKAFHFLRPVIQGCADADEGLTEAELQSTLDFCTNV
FAPTQPETHEAAIQRLGSLADPDGPLLDTDTIDKLTGTDMEAKHALWKINARKPLHSMYDCK
RNFGTEIINGFYVKMEQGVLGLVRT
>B38
MSDSKAPNVVVLGGGSAGWITACLLHRQWSEKGGSVTVVESPEIGIIGVGEGSTPQLKAFFD
QLGIAESEWMPACNATYKLGIRFNGWSERAGFESYYHPFPGPIDLHSEPGFIGNCMLARHQID
VAAHPDDWFLATHLSEAGKSPIAAENFPFAPSYGYHFDAYALGAFLRDWAVKRGVVHRPLK
VTAVERADDGDIAALLCEGGERIEGDLFVDCSGFRAMLAEQELGATFLPFAENLFNDSAVVL
PTGHGAPFKPQTEATAMRAGWRWSIPLTTRVGNGYVYSSKYISNEDAEAELRGALGLAGDG
PEARFLKMKVGRLENSWTRNCLAVGLSQGFLEPLEATALHIVIVTALEFAQAYEQGGYGPQH
RDAFNASIAARYEGIRDYIVAHYRVNQRSDTQYWRDNAANDRLSNGLKTMMTAWFRHQDI
HAANDEAYGGKRYYANASWHCLFAGYGTFPPQERMEAVPEGIRPADPAEARAMLEACAQN
FPSYDPIAS
>BhaA
MSVEDFDVVVAGGGPGGSTVATLVAMQGHRVLLLEKEVFPRYQIGESLLPATVHGVCRML
GISDELANAGFPIKRGGTFRWGARPEPWTFHFGISAKMAGSTSHAYQVERARFDEMLLNNAK
RKGVVVREGCAVTDVVEDGERVTGARYTDPDGTEREVSARFVIDASGNKSRLYTKVGGSRN
YSEFFRSLALFGYFEGGKRLPEPVSGNILSVAFDSGWFWYIPLSDTLTSVGAVVRREDAEKIQ
GDREKALNTLIAECPLISEYLADATRVTTGRYGELRVRKDYSYQQETYWRPGMILVGDAACF
VDPVFSSGVHLATYSALLAARSINSVLAGDLDEKTALNEFELRYRREYGVFYEFLVSFYQMN
VNEESYFWQAKKVTQNQSTDVESFVELIGGVSSGETALTAADRIAARSAEFAAAVDEMAGG
DGDNMVPMFKSTVVQQAMQEAGQVQMKALLGEDAEPELPLFPGGLVTSPERMKWLPHHP
A
>Bmp2
GSHMNGFTHYDVVIIGSGPAGSLCGIECRKKGLSVLCIEKEQFPRFHIGESLTGNAGQIIRDLG
LAEDMDAAGFPDKPGVNVIGSLSKNEFFIPILAPTWQVRRSDFDDMIKRKAVEHGVEYKLGM
VTDVIKDGEKVVGALYKADGVEHQVRSKVLVDASGQNTFLSRKGVAGKRQIEFFSQQIASF
AHYKGVERDLPPFSTNTTILYSKQYHWSWIIPISPDTDSLGVVIPKDLYYKECKNPDDAIAWG

MDHISPELKRRFKNAERQGDSQSMADFSYRIEPFVGDGWMCIGDAHRFLDPIFSYGVSFAMK
EGIRAAEAIAQVVAGQDWKAPFYAYRDWSNGGQQIAADLIRYFWIYPIFFGYQMQNPDLRD
EVIRLLGGCCFDCEGWKAPAIFRNAIEEYDRKQMAS
>Bmp5
MRKKIAVIGAGLSGIAAIKQLTDGGHEVVCFEKAESFGGVFADKKIYEDLHLTISNYFMAYSD
YVPSQQKLKFWSKKEYVNYLGEYLARFELGQYIHYDHEVRKVEKQAGKWQVTTKHGMAE
QTDTFDMVAVCSGHFQKPKMPELAGLDMFEGEIEHSNDYRDKHKYAGKRVLCVGLGESSA
DITSEISEVASKCILSLRRYPAVAPRYMAFQEDPYFTIDTSWLTSRIVNKLPHRYHGGITKGIFT
KYVNSRNDHVRIRGEWLKKSGPSHHQAVTKNERLFRPIADGKVVPNIGGIVRFEKNAVVFQD
GTREEIDAVVFCTGYQLSFPFLDVSISNMRDLYKQMFIPEMGDSLSFIGFVRPQQGGIPVIAEM
QCRYLSQLASGEKSLPPLSEMVDIIKYDTEHWQTEYKITPHVASLVNYCHYMDSMAKLVGC
MPEIPSLFKDPLLRVKLLHNPQFAAQYRLDGPNKMTHTARSFLLGFPNISSWPRIIHFELALITQ
KLLSRLRLDGLRELSK
>BrvH
MDEIDDPRIRSVVIVGGGTAGWMTAAALVQHFRTAPLKITVVESSDIGTIGVGEATIPTIRRFY
GQLGLRDDDVMRATQATCKLGIRFLDWSGPGSDFIHPFGLYGQDVKGIGFHHYWLKQRRAG
DAAPLAAYSLGAALAAGGKFTLPSPHPPSQLSVFDWALHLDAGLFAQHLRAYAEAGGCARI
DARIRSVELRPEDGFVRALTLDDGREVEGDLFVDCSGFKGLVIGEALGVGFEDWGRWLPCD
AAYAVQSENRPGDAPAPFTRVTARSAGWQWGIPLRHRAGNGLVFSSAHLSDDQALAELMPH
LLGDPLTEPRRIPFRPGRRSQAWAKNCVAIGLSSGFLEPLESTSIALIETGIERLKALFPDRRFA
QPILDEFNDQTAREMERVRDFIILHYKLNRRTDTDFWRDCREMPVPETLERKIALWTARGQF
VRYRWEMFHPASWLAIYDGFGLYPDHHDPAVDAMDPAYLARSLAEMRANIADLVARTPEH
AQFLAGLDPAASAA
>ChlA
MDPTDIQSDLKDKLFGARKNCSTPSGSTMEAVGNKHYDVVVIGAGIAGLSAVRHLLLKIPEL
RANRVAVIDPRSSIRNNLLEDYKVGESTVEISGMFFARELELQDYLIENQPPKFSLQFHWPKK
VDKTDSMDDYHSTWAVKNPDIQAFQLNRSKMEKDLLQMVIDQGAVYYHGRVKDVDIGMG
DAVKTIDIQMLSEEVNFDDQKPLERINITTDYIADASGRNFIIGTRTDNVLKDPKHLFGLQNAS
TWLRVKNTDKKYFDFNDQSVTSSWYYATNHFFGPGYWIWMIPIERATHDFSIGVSYHRDKY
DPSEFNSLDKFMAFLETNQKVLYNIIKSGEIVDFHRWPRLAHTSKVFVSEDNWCVLGDAAAIF
DPFYSTGMVMIAMEVECLTELTKYRLSGDKCATKTRQQAYDKLIRAITQTNCHLIKKHSDHL
GNASIMSWRIYLESLSYFGILLPAYIGKYHLCPIFGHEFADNNWRLIEFRDRLLNLMDYIGQQG
QNVGFMDNHRGGQLIGSFCPTSSWDYEHCISMCMYGHKRLNLHKYMIWTHFYQMAGVIKM
LYRAFGARLLLNGDFQWMMTHTMKTMGFHSVKSWVHYLSNISNPVTDDYYDRNQISFKEY
QYTGEAVPWESSM
>Clohal

MSENQEYDVIVIGGGPGGSMVSSLLADGGKKVLVLEVAKFPRYHIGESLLLGTVDLLDKIGV
REKLEAGDYIKKYGVEWVWGEQREPWKVDFRNAVSVTHDYTYQVERGPFDKMLLDNARE
HGVDVREQHRVTNFSIDDNGRSTVNYRRTDTGETGTATARWLVDASGQGGLVTKRLHTQE
WDPYLKNMAVWSYWKGVKRSEGEDAGNIFLPTFDDGWWWCIPLRDDITSIGAVVDRESLN
ALKSTRVRQYYLDSIEKTPELASRTENAELVDDMHVQRDWSYIYDRFCGDGYIAIGDAACFI
DPLFSTGVHLAMLSGFLAATVVNTILDKPELDTARMLQFYEGAYRKEFARLRDQVYFLYAG
NKGSKESYFWHARSHFGVPSIEPEKAFVSLIAGAFAHRAWYNHYAKHLDVSADLKKSVESM
FNGAGIDLDTPLIASACGSVVNDFAVDGRYLREARSLVHTNGASIAYTPAVRAAVEHADGKR
TGREIIEIITDRGIDSEDRARSIVHEVISYGFVEPKA

>CndH

MSTRPEVFDLIVIGGGPGGSTLASFVAMRGHRVLLLEREAFPRHQIGESLLPATVHGICAMLG
LTDEMKRAGFPIKRGGTFRWGKEPEPWTFGFTRHPDDPYGFAYQVERARFDDMLLRNSERK
GVDVRERHEVIDVLFEGERAVGVRYRNTEGVELMAHARFIVDASGNRTRVSQAVGERVYSR
FFQNVALYGYFENGKRLPAPRQGNILSAAFQDGWFWYIPLSDTLTSVGAVVSREAAEAIKDG
HEAALLRYIDRCPIIKEYLAPATRVTTGDYGEIRIRKDYSYCNTSFWKNGMALVGDAACFVD
PVFSSGVHLATYSALLVARAINTCLAGEMSEQRCFEEFERRYRREYGNFYQFLVAFYDMNQD
TDSYFWSARKIINTEERANEAFVRLIAGRSNLDEPVFQSVAKDFFTEREGFGAWFGGLVTSM
AKGDGGGLMVGEGATDATESTGFAPENFMQGFTREITELQHLAMFGEDRGPETPLWSGGLV
PSRDGLAWAVESGEDAAG

>CmlS

MTRSKVAIIGGGPAGSVAGLTLHKLGHDVTIYERSAFPRYRVGESLLPGTMSILNRLGLQEKI
DAQNYVKKPSATFLWGQDQAPWTFSFAAPKVAPWVFDHAVQVKREEFDKLLLDEARSRGI
TVHEETPVTDVDLSDPDRVVLTVRRGGESVTVESDFVIDAGGSGGPISRKLGVRQYDEFYRN
FAVWSYFKLKDPFEGDLKGTTYSITFEDGWVWMIPIKDDLYSVGLVVDRSKSAEVREQGAD
AFYSSTLAKCAKAMDILGGAEQVDEVRIVQDWSYDTEVFSADRFFLCGDAACFTDPIFRRSV
LFSQGVHLASQSAVSAAAAIDRITRHGDEKDAVHAWYNRTYREAYEQYHQFLASFYTFASF
TEPDSEFWRKRRITESDDDRLTRKKWFESLAGNGPEDPSGTVASFRDRASTMIAIGRHQRPEL
SDDFSEAELNPARVRWISDLTKRLNSITRFKWTGGKAVLKQHYRVEPIGFRLEQREVLANGE
GLDMAQYPMDDEARQIFQDLAEEEFGYKTLVKRLGAVGRQELSTQIVVRLMEAGLLTGYDA
QGEKVFVQGRLHFGGVGVEYEV

>HalB

MSSAPAAGIDPAVSHARTFDVAILGSGIAGSMLGAILARNGARVLLVDASTHPKFAVGESTIP
YTLVALRTIAERYDVPEIKTLATFTNCTKVIGPQFGVKKHFGFLLHREGEPQDPREVNQFDTP
GLLHEASHMYRQATDAYLFHAAVKYGCVPRQNFRVVDVDFDDAGVTISGGDGEQYRARYV
VDASGFRSPLAEKFALRENPCRMKHHSRSLWNHMVNVPRTDALFDHSAADTPPVPWYEGT
VHHMFDRGWFWVIGFDNHPASRNPLCSVGLTLDERKYPKDPSLTPQQEFESMAARYPDIAR
QFAGANPVREWVSTDRLQYSSKQTVGDRWCLLSHAAGFVDPLFSRGLSNTAEAINALSWRLI

AAIKDDDFSQERFEYVDRLQQGLFDYNDALVNASFIAFNHYDLWTAVFRIWAWGSNAGTFF
LQEALTKFLRDGRDEHFRAQEDVPHLGLYWPSHDGYKKLFDEMIAQTDAYEQGLVTGKQA
ADALYDILVHADFVPKHFGFADRNRRFLHPTPNVLRKTVRWAMREADPDVRRLMIGTGKEA
VKRKLTGRRIF
>HalX
SAQRADVVITGGGLAGLSLALQLRQRDPALAITVLERRAHPVREAAFKVGESTVEIGAHYFA
DVLGLREHLEAEQIRKFGFRFFFSDKRDDIDRCTELGVSKILPTPSWQIDRGRFENFLGERARA
QGIVFLDSCSVKGVDLSDDDASDHRVRYERGGEAGTLDARWVVDASGRAGLLKRKLGLTQ
DNAHDANAVWWRVEGLIDPNAWSQDSSWLQRCTPPDRWRSTNHMCGPGYWFWLIPLSSG
AHSLGIVCDAAMHPLETMNTHDKAMAWLRTHQPQVAATLDQTDYRLQDFLFLRNFSYGCK
QVFSTQRWALTGEAGVFLDPFYSPGSDFIAISNTYICELIGRDRAGRSLSPYVELYEQLYFSFY
ENTLSLYQDQYALFGDAQVMPVKVIWDYTYYWSLLAPLFCSGRIADLGLLARMKADFFYAR
DMNLAMQRVLHDWGQHNTALGTVTGDGRLLDQYLIGWFNELNGALHDTLDDEAFAARIH
ANIARMAVLAREILEQARQRHPALPDHGLAALTANAVGDPILTAAWYADAA
>HalY
MVTDFDIGIIGGGPGGTTAASYLARAGLSVAVFEGELFPREHVGESLVPATTPVLREIGALAK
LDEAGFPRKYGAAWTSPSGARASDTFGMADLAFAEDEDVLGGQSYTYHVDRGRFDQLLLE
HSSTLGAKVFEGARVRRVDFDGPHPRIVHASGGSESRTGVRMVIDASGRGTLLGRQLGLKVT
DPTFDQYAVHGWFTGLDREAIAGRDALADYLFVHFLPAEDSWVWQIPITDTITSVGVVSRKS
RLRQAGGDREAFFWGTLEHRPELAAALRACTPVRPLKVDGDFSYAMRQITGDGWAMVGDA
ARFVDPIFSSGVGIAMNGARLLSQDVLAAAEAGDFARQRFAEYEKKQRRGLTNWYNFIAVY
YRLNILFTVFVQDPRYRLDVLRMLQGNVYETDHPPVLTTMQGVVETVESNPDHLWHPHLGS
LRAPAAAPLF
>HrmQ
MSDFDYDIGIIGGGPAGSTMASYLAKAGISAIVLESAEFPRPHVGESLVTATTPVLQEIGALAK
VDAAGFPKKYGAAWTSAAKETVPEMGFTGMTQGFRLASVEFQERDQPGVDQAYTYHVDR
GKYDQLLLQHAAELGARVRERTRVQDVEFTDDGVVLTAVGDEGTERLRVRMVVDASGRQT
MLGRKKKLKVPDPVFNQYAIHAWFDGLDRKALADDPEQEDFIFIHFLPVSDTWVWQIPISET
TTSVGVVTQKAKVKSSGQEREEFFWECLGTRPELRAALEKAERVREFKTEGDYSYGMKEVV
GDRWALIGDAARFVDPIFSSGVSVAMNSARLLAKDIVSAVERDDFSKKSFGDYERVLRRGVS
SWYEFISIYYRLNILFTAFVQDPRYRLDVLKMLQGDVYGEEEPKALTRMREIVRRVEENPDHL
WHPYLGTLRAPAAAPLF
>KrmI
MASGACTSHRSDRQVPHEHTAIKSICVVGGGTAGYLSALTLRRRMPHLKISLLESSRVPIIGVG
EATTPSLPALLHGVLDMDIVDFFTQVKPTFKMGIRFEWGGTTPEHYFNYPFGAHGQLLEAMT
YQGHIRDYSLGSMLMSADKMAIFRTGDGAYESRLHYGAISYAYHIDNQRFVSWLQREAKRA
GLHHIDANIQDVVTDDSGQNVVALLTDDGARHEYDFYVDCSGFRSLLLEKALDSEWVPYDQ

TLFTDRAVTANVPHGGHIKPYTVAETMDHGWCWNIPMREDDHRGYVFSSAFCDEETAIKEM
QAKNPGMSDTKLVTFRSGRHRHFWKGNVVAIGNSYAFVEPLESTGVHMIAEEILAFIGNFPQ
STHDDANRAALNRYMAAYWDELRWFLGLHFRFNTKLDTPFWKACRADVDVSGFDDCLAT
YRKCAPLSYRNHYLTFNRLWGDHGRDVLLMGQQVPANFLPPRESKQQWLRRVELARQAVE
MAVDQAEAIRLLESNPDVLRQLVTDDGAWIHTVGELFSSETALYSDVYAARLGHMKKPAGF
GGAQNVILPSTL

>KtzQ

MDDNRIRSILVLGGGTAGWMSACYLSKALGPGVEVTVLEAPSISRIRVGEATIPNLHKVFFDF
LGIAEDEWMRECNASYKAAVRFVNWRTPGDGQATPRRRPDGRPDHFDHLFGQLPEHENLPL
SQYWAHRRLNGLTDEPFDRSCYVQPELLDRKLSPRLMDGTKLASYAWHFDADLVADFLCRF
AVQKLNVTHVQDVFTHADLDQRGHITAVNTESGRTLAADLFIDCSGFRSVLMGKVMQEPFL
DMSKHLLNDRAVALMLPHDDEKVGIEPYTSSLAMRSGWSWKIPLLGRFGSGYVYSSQFTSQ
DEAAEELCRMWDVDPAEQTFNNVRFRVGRSRRAWVRNCVAIGVSAMFVEPLESTGLYFSY
ASLYQLVKHFPDKRFRPILADRFNREVATMYDDTRDFLQAHFSLSPRDDSEFWRACKELPFA
DGFAEKVEMYRAGLPVELPVTIDDGHYYGNFEAEFRNFWTNSNYYCIFAGLGFLPEHPLPVL
EFRPEAVDRAEPVFAAVRRRTEELVATAPTMQAYLRRLHQGT

>KtzR

MTAAYLKTAFGDRLSITVVESSRIGTIGVGEATFSDIQHFFQFLNLREQDWMPACNATYKLGI
RFENWRHVGHHFYQPFEQIRPVYGFPLTDWWLHDAPTDRFDTDCFVMPNLCEAGRSPRHLD
GTLADEDFVEEGDELANRTMSEHQGKSQFPYAYHFEAALLAKFLTGYAVDRGVEHVVDDV
LDVRLDQRGWIEHVVTAEHGEIHGDLFVDCTGFRGLLLNKALGVPFVSYQDTLPNDSAVAL
QVPLDMQRRGIVPNTTATAREAGWIWTIPLFGRVGTGYVYAKDYLSPEEAERTLREFVGPAA
ADVEANHIRMRIGRSQESWRNNCVAIGLSSGFVEPLESTGIFFIHHAIEQLVKHFPAADWNPK
SRDMYNSAVAHVMDGIREFLVIHYRGAARADNQYWRDTKTRPLPDGLAERIECWQTQLPDT
ETIYPYYHGLPPYSYMCILMGGGAIRTPASAALALTDQGAAQKEFAAVRDRAAQLRDTLPSH
YEYLARMRGLDV

>MalA'

MAPTPKYTFTERAAAGNLSDAEILNSNNPTGSELPDESDVVVGGAGIHGLIYALHASKYKPN
NLKISVIEKNTRPGYKIGESTLPIFYTWCKLHGISAAYLLRLFGLKDGLCFYFLDRENQGQYTD
FCSVGAPGLVLASLQIERPMSELLFTILAQRNGVNVYHGREVDFKSTVVQGGGQGNKIAVSR
GKYDSTPKTIDSALFVDATGRFRQFCSKKAPRHRFDGWNCNAFWGYFTAPKDESKIPFDLYE
GDHTNHLCFPEGWVWVIRLPSWEGSLIANLMDMVTYILECADAGVPGDELPSSEELARMFG
LKFQWVTSIGFAVRNDVKYPEDLSAYGTREAEQKFNYFVQKYELLQQFMSNFELIENLYGPG
TTWFIRKTLAYQSPVVSGPGWLAIGDACGFTNPLYSPGINVGMSTSTWAAQLSHRIVEIGKSA
PADAAESSIRKLLVPYDDYCKSLVPALEQMNRFNYVCYRDTRLGPQVACLWQFFAGIERYLS
DVNIETFAHYAIKWVWGAMVPEYQQVAQKCIEHIETVPLDERLPDAMVDELLAFSNRIKSAA

VAADDFSLRWDAILRSFDRSLNFVEGKTSRDIYTRQCSGCGAWLQLRPDWKKCHSCGLLGT
EPQTAVTFDPPLTAEEEALLYAAWNTAPKYDPSKELKLPTPTRPAA
>MibH
MARSEESNTLARLFDVLGDDAAAAREWVTEPHRLIASNERLGTAPEAPADDDPGAIRTVGVI
GGGTAGYLTALALKAKRPWLDVALVESADIPIIGVGEATVSYMVMFLHHYLGIDPAEFYQH
VRPTWKLGIRFEWGSRPEGFVAPFDWGTGSVGLVGSLRETGNVNEATLQAMLMTEDRVPV
YRGEGGHVSLMKYLPFAYHMDNARLVRYLTELAARRGVRHVDATVAEVRLDGPDHVGGLI
TTDGRRLHYDFYVDCTGFRSLLLEKALGIPFESYASSLFTDAAVTGTLAHGGHLKPYTTATT
MNAGWCWTIPTPESDHLGYVFSSAAIDPDDAAAEMARRFPGVTREALVRFRSGRHREAWRG
NVMAVGNSYAFVEPLESSGLLMIATAVQILVSLLPSSRRDPLPSDAANQALAHRWDAIRWFL
SIHYRFNGRLDTPFWKEARAETDISGIEPLLRLFAAGAPLTGRDSFTRYLADGAAPLFYGLEG
VDTLLLGQEVPARLLPPREPPEQWRARAAAARSLASRGLRQSEALDAYAADPCLNAELLSDS
DSWAGERVAVRAGLR
>Mpy16
MEPQFDVGIIGGGPAGSTTASYLARAGLKVALFESDNFPREHVGESLVPATTPVLVDIDAFDK
VEAAGFPKKFGAAWTSADSGPSDKMGFTGLDHDFRAAEIMFNERTQSGVHKDYTFHVDRG
QFDLLLLKHAEEQGAKVHQGVRVNRVNFDGAFPVLETSVAGQRAKVPVKMVVDASGRRTQ
LGSQLKVKEKDPVFNQYAIHTWFDNFDRKALAVDQSQSDFIFIHFLPVIDTWVWQIPITDTITS
VGVVTQKERLKASKDDLEKFFWDTLGSRPELHKALKESEQVRPLKTEGDYSYALTKVCGDN
FLMVGDAARFVDPIFSSGVSVALNSARIASADIIAAHRAGDYSKKRFDTYESMLRRGVNNWY
EFISIYYRLNILFTAFVQDPRYRIDVLKMLQGDVYDDEEPKALAAMREIVKAVEEDPNHLWH
PFLGSLKAPSAKAMF
>Pia-2
MKHGGMASGAIERVVIVGGGTAGWMAAAALGAYLAGAGTRITLIESSEIGTIGVGEATIPTIR
RFYAALGMTDAEVMRACEATAKLGIRFVDWKPGTSFVHPFGRFGQDLRGIDFHQYWQKAR
QAGRAAPLEEYSLGAMLAREGHASVPLPNPPSQLSVFDWALHFDAARFAAHMRAYAERAG
VARIDARITDVTLHGETGFIEAVTLDSGERVAGDLFVDCSGFRGLLIGEALGTPYRDWSHWLL
CDGAFAVQSERVGEPPSCTTVTARTAGWQWRIPLRSREGNGLVFASDFQGDDEARAELLANI
PGAPTMEPRRLRFTPGRREVAWARNCVSLGLASGFLEPLESTSIALIETGIERLKQLFPDKGFD
PAVIAEYNAQSAEEMERVRDFILLHYHLSTRDGPFWQACREMTLPDSLAAKLELWRARGAF
LRYRWEMFSPASWLAIYGGFEHLPERLDPGVAAVGTDELAQGLEQMRAAIARTVADTPTHS
EFLATVDGAAAEPMRTSA
>PltA
MSDHDYDVVIIGGGPAGSTMASYLAKAGVKCAVFEKELFEREHVGESLVPATTPVLLEIGVM
EKIEKANFPKKFGAAWTSADSGPEDKMGFQGLDHDFRSAEILFNERKQEGVDRDFTFHVDR
GKFDRILLEHAGSLGAKVFQGVEIADVEFLSPGNVIVNAKLGKRSVEIKAKMVVDASGRNVL
LGRRLGLREKDPVFNQFAIHSWFDNFDRKSATQSPDKVDYIFIHFLPMTNTWVWQIPITETITS

VGVVTQKQNYTNSDLTYEEFFWEAVKTRENLHDALKASEQVRPFKKEADYSYGMKEVCGD
SFVLIGDAARFVDPIFSSGVSVALNSARIASGDIIEAVKNNDFSKSSFTHYEGMIRNGIKNWYE
FITLYYRLNILFTAFVQDPRYRLDILQLLQGDVYSGKRLEVLDKMREIIAAVESDPEHLWHKY
LGDMQVPTAKPAF

>PltM

MNQYDVIIIGSGIAGALTGAVLAKSGLNVLILDSAQHPRFSVGEAATPESGFLLRLLSKRFDIP
EIAYLSHPDKIIQHVGSSACGIKLGFSFAWHQENAPSSPDHLVAPPLKVPEAHLFRQDIDYFAL
MIALKHGAESRQNIKIESISLNDDGVEVALSNAAPVKAAFIIDAAAQGSPLSRQLGLRTTEGLA
TDTCSFFTHMLNVKSYEDALAPLSRTRSPIELFKSTLHHIFEEGWLWVIPFNNHPQGTNQLCSI
GFQFNNAKYRPTEAPEIEFRKLLKKYPAIGEHFKDAVNAREWIYAPRINYRSVQNVGDRFCLL
PQATGFIDPLFSRGLITTFESILRLAPKVLDAARSNRWQREQFIEVERHCLNAVATNDQLVSCS
YEAFSDFHLWNVWHRVWLSGSNLGSAFLQKLLHDLEHSGDARQFDAALEAVRFPGCLSLDS
PAYESLFRQSCQVMQQAREQARPVAETANALHELIKEHEAELLPLGYSRISNRFILKV

>PrnA

MNKPIKNIVIVGGGTAGWMAASYLVRALQQQANITLIESAAIPRIGVGEATIPSLQKVFFDFLG
IPEREWMPQVNGAFKAAIKFVNWRKSPDPSRDDHFYHLFGNVPNCDGVPLTHYWLRKREQG
FQQPMEYACYPQPGALDGKLAPCLSDGTRQMSHAWHFDAHLVADFLKRWAVERGVNRVV
DEVVDVRLNNRGYISNLLTKEGRTLEADLFIDCSGMRGLLINQALKEPFIDMSDYLLCDSAVA
SAVPNDDARDGVEPYTSSIAMNSGWTWKIPMLGRFGSGYVFSSHFTSRDQATADFLKLWGL
SDNQPLNQIKFRVGRNKRAWVNNCVSIGLSSCFLEPLESTGIYFIYAALYQLVKHFPDTSFDPR
LSDAFNAEIVHMFDDCRDFVQAHYFTTSRDDTPFWLANRHDLRLSDAIKEKVQRYKAGLPL
TTTSFDDSTYYETFDYEFKNFWLNGNYYCIFAGLGMLPDRSLPLLQHRPESIEKAEAMFASIR
REAERLRTSLPTNYDYLRSLRDGDAGLSRGQRGPKLAAQESL

>PrnC

MTQKSPANEHDSNHFDVIILGSGMSGTQMGAILAKQQFRVLIIEESSHPRFTIGESSIPETSLMN
RIIADRYGIPELDHITSFYSTQRYVASSTGIKRNFGFVFHKPGQEHDPKEFTQCVIPELPWGPES
HYYRQDVDAYLLQAAIKYGCKVHQKTTVTEYHADKDGVAVTTAQGERFTGRYMIDCGGPR
APLATKFKLREEPCRFKTHSRSLYTHMLGVKPFDDIFKVKGQRWRWHEGTLHHMFEGGWL
WVIPFNNHPRSTNNLVSVGLQLDPRVYPKTDISAQQEFDEFLARFPSIGAQFRDAVPVRDWV
KTDRLQFSSNACVGDRYCLMLHANGFIDPLFSRGLENTAVTIHALAARLIKALRDDDFSPERF
EYIERLQQKLLDHNDDFVSCCYTAFSDFRLWDAFHRLWAVGTILGQFRLVQAHARFRASRN
EGDLDHLDNDPPYLGYLCADMEEYYQLFNDAKAEVEAVSAGRKPADEAAARIHALIDERDF
AKPMFGFGYCITGDKPQLNNSKYSLLPAMRLMYWTQTRAPAEVKKYFDYNPMFALLKAYIT
TRIGLALKK

>PyrH

MERRKRERLGSLGRPTKKELRMIRSVVIVGGGTAGWMTASYLKAAFDDRIDVTLVESGNVR
RIGVGEATFSTVRHFFDYLGLDEREWLPRCAGGYKLGIRFENWSEPGEYFYHPFERLRVVDG

FNMAEWWLAVGDRRTSFSEACYLTHRLCEAKRAPRMLDGSLFASQVDESLGRSTLAEQRAQ
FPYAYHFDADEVARYLSEYAIARGVRHVVDDVQHVGQDERGWISGVHTKQHGEISGDLFVD
CTGFRGLLINQTLGGRFQSFSDVLPNNRAVALRVPRENDEDMRPYTTATAMSAGWMWTIPL
FKRDGNGYVYSDEFISPEEAERELRSTVAPGRDDLEANHIQMRIGRNERTWINNCVAVGLSA
AFVEPLESTGIFFIQHAIEQLVKHFPGERWDPVLISAYNERMAHMVDGVKEFLVLHYKGAQR
EDTPYWKAAKTRAMPDGLARKLELSASHLLDEQTIYPYYHGFETYSWITMNLGLGIVPERPR
PALLHMDPAPALAEFERLRREGDELIAALPSCYEYLASIQ
>Rdc2
MSVPKSCTILVAGGGPAGSYAAAALAREGNDVVLLEADQHPRYHIGESMLPSLRPLLRFIDLE
DKFDAHGFQKKLGAAFKLTSKREGYTDFIAAHGPRGYSWNVVRSESDEILFNHARESGAQAF
QGIKINAIEFEPYEEEYPNGEKVANPGKPTSAKWSSKDGSSGDIAFKYLVDATGRIGIMSTKYL
KNRHYNEGLKNLAIWGYYKNNIPWAQGTPRENQPFFEGMRDGAGWCWTIPLHNGTVSVGA
VMRKDLFFEKKKSLGENATNTQIMAECMKLCPTIGELLAPAELVSDIKQATDYSYSATAYAG
PNFRIVGDAGCFIDPFFSSGHHLAVAGALAAAVSINASIKGDCTEYEASRWHAKKVDEGYTL
FLLVVMAALKQIRMQENPVLSDVDEDGFDRAFQFLRPVIQGSGSSEAVKKFTKEDVAQTIDF
AVHALNNMAELDMDIPEHMINGDKEGENGVTNGNNGAAKTAGLASNMEKLTNDEEKVLN
GLRILGKAAPGGTLADFEGTAIDGLMPRLEHGKLGLNKV
>RebH
MSGKIDKILIVGGGTAGWMAASYLGKALQGTADITLLQAPDIPTLGVGEATIPNLQTAFFDFL
GIPEDEWMRECNASYKVAIKFINWRTAGEGTSEARELDGGPDHFYHSFGLLKYHEQIPLSHY
WFDRSYRGKTVEPFDYACYKEPVILDANRSPRRLDGSKVTNYAWHFDAHLVADFLRRFATE
KLGVRHVEDRVEHVQRDANGNIESVRTATGRVFDADLFVDCSGFRGLLINKAMEEPFLDMS
DHLLNDSAVATQVPHDDDANGVEPFTSAIAMKSGWTWKIPMLGRFGTGYVYSSRFATEDEA
VREFCEMWHLDPETQPLNRIRFRVGRNRRAWVGNCVSIGTSSCFVEPLESTGIYFVYAALYQ
LVKHFPDKSLNPVLTARFNREIETMFDDTRDFIQAHFYFSPRTDTPFWRANKELRLADGMQE
KIDMYRAGMAINAPASDDAQLYYGNFEEEFRNFWNNSNYYCVLAGLGLVPDAPSPRLAHM
PQATESVDEVFGAVKDRQRNLLETLPSLHEFLRQQHGR
>StaI
MDGLSGDPLPSTIAKDSIEGWWEMSVETFDVVVAGGGPGGSTVAALVAMQGHRVLLLEKE
VFPRYQIGESLLPSTVHGVCRLLGVTDELAAAKFPVKRGGTFRWGARPEPWTFSFAVSPRMT
GPTSFAYQVERARFDEILLNNAKSKGVTVREGCSVTAVVEEGDRVTGLRYTDSDGGEHEVG
ARFVIDASGNKSRLHASVGGTREYSEFFRSLALFGYFENGKRLPEPYSGNILSVAFDSGWFWY
IPLSDTLTSVGVVVRREMAEKIQGDREKAYNALIAECPLIAEYLSDARRVTTGNYGELRVRK
DYSYHQTKFWRPGMILVGDAACFVDPVFSSGVHLATYSALLAARSINSVLAGDLDEKTAFDE
FEGRYRREYGVFYEFLVSFYQMNVNEESYFWHAKKVTNNEHTEFESFVDLVGGVASGETAL
AASRLAERSAEFAGAVDQMAGGDDGDMVPLFKSHVVKQVMQEGGQVQMKAVLGEDAAP
EEPVFPGGLVSSPDGMKWLPYRG

>SttH

MNTRNPDKVVIVGGGTAGWMTASYLKKAFGERVSVTLVESGTIGTVGVGEATFSDIRHFFEF
LDLREEEWMPACNATYKLAVRFQDWQRPGHHFYHPFEQMRSVDGFPLTDWWLQNGPTDR
FDRDCFVMASLCDAGRSPRYLNGSLLQQEFDERAEEPAGLTMSEHQGKTQFPYAYHFEAAL
LAEFLSGYSKDRGVKHVVDEVLEVKLDDRGWISHVVTKEHGDIGGDLFVDCTGFRGVLLNQ
ALGVPFVSYQDTLPNDSAVALQVPLDMEARGIPPYTRATAKEAGWIWTIPLIGRIGTGYVYA
KDYCSPEEAERTLREFVGPEAADVEANHIRMRIGRSEQSWKNNCVAIGLSSGFVEPLESTGIFF
IHHAIEQLVKHFPAGDWHPQLRAGYNSAVANVMDGVREFLVLHYLGAARNDTRYWKDTKT
RAVPDALAERIERWKVQLPDSENVFPYYHGLPPYSYMAILLGTGAIGLRPSPALALADPAAA
EKEFTAIRDRARFLVDTLPSQYEYFAAMGQRV

>Tar14

MSVSGSERSAEGNRKKRVVIVGGGTAGWMTASYLTAAFGDRVDLTVVESAQIGTIGVGEAT
FSDIRHFFEFLRLEESDWMPECNATYKLAVRFENWREPGHHFYHPFEQMSSVDGFPLSDWW
LRNPTTSRFDKDSFVMTSLCDAGVSPRYLDGSLIDQDFVEQERDDDSARSTIAEYQGAQFPYA
YHFEAHLLAKYLTGYATRRGTRHIVDNVVDVALDERGWISHVRTEEHGDLEADLFVDCTGF
RGLLLNKALGEPFVSYQDTLPNDSAVALQVPLDMEREPIRPCTTATAQEAGWIWTIPLISRVG
TGYVYASDYTTPEQAERVLRDFVGPAAADVPANHIKMRIGRSRRSWVNNCVGVGLSSGFVE
PLESTGIFFIHHAIEQIVKYFPSGGAGDDRLRELYNRSVGHVMDGVREFLVLHYRSAKRADNQ
YWKDTKTRTVPDSLAERIEFWKHKVPDAETVYPYYHGLPPYSYNCILLGMGGIDVNYSPAL
DWANEKAALAEFERIRVKAEKLVQELPTQNEYFAAMRAGR

>Tcp21

MTSLSSDDSIDGWWKMPVEEFDVVVAGGGPAGSTVATLVAMQGHRVLLLEKETFPRYQIGE
SLLPSTVHGVCRMLGVSDELAAAGFPVKRGGTFRWGARPEPWTFSFAVSPLMNGPTSFAYQ
VERARFDEILLRNAQRKGVDVREGCSVTGVIEGDERITGVRYTDPDGGEHEVSARFVVDASG
NKSRLYSQVGGVRNYSEFFRSLALFGYFENGKRLPEPYSGNILSVAFDSGWFWYIPLSDTMTS
VGAVVRREMAEKIQGDREKALQALIAECPLISDYLADARRITTGRYGELRVRKDYSYHQTSY
WRPGMILVGDAACFVDPVFSSGVHLATYSALLAARSINSVLAEDLDEKTALSEFEARYRREY
GVFYEFLVSFYQMNVNEESYFWQAKKVTNNKHTEIESFVELIGGVSSGEAALATAGRIAERS
AEFAAAVDQMAASADGNMVPMYKSEVVKQVMQEGGQVQMKAVLGADAEPELPLFPGGL
VVSPDGMKWLPYRPA

>Thal

MDNRIKTVVILGGGTAGWMTAAYLGKALQNTVKIVVLEAPTIPRIGVGEATVPNLQRAFFDY
LGIPEEEWMRECNASYKMAVKFINWRTPGEGSPDPRTLDDGHTDTFHHPFGLLPSADQIPLSH
YWAAKRLQGETDENFDEACFADTAIMNAKKAPRFLDMRRATNYAWHFDASKVAAFLRNF
AVTKQAVEHVEDEMTEVLTDERGFITALRTKSGRILQGDLFVDCSGFRGLLINKAMEEPFIDM
SDHLLCNSAVATAVPHDDEKNGVEPYTSSIAMEAGWTWKIPMLGRFGSGHVYSDHFATQDE
ATLAFSKLWGLDPDNTEFNHVRFRVGRNRRAWVRNCVSVGLASCFVEPLESSGIYFIYAAIH

MLAKHFPDKTFDKVLVDRFNREIEEMFDDTRDFLQAHYYFSPRVDTPFWRANKELKLADSIK
DKVETYRAGLPVNLPVTDEGTYYGNFEAEFRNFWTNGSYYCIFAGLGLMPRNPLPALAYKP
QSIAEAELLFADVKRKGDTLVESLPSTYDLLRQLHGAS
>Th-Hal
LNNVVIVGGGTAGWMTASYLKAAFGDRIDITLVESGHIGAVGVGEATFSDIRHFFEFLGLKE
KDWMPACNATYKLAVRFENWREKGHYFYHPFEQMRSVNGFPLTDWWLKQGPTDRFDKDC
FVMASVIDAGLSPRHQDGTLIDQPFDEGADEMQGLTMSEHQGKTQFPYAYQFEAALLAKYL
TKYSVERGVKHIVDDVREVSLDDRGWITGVRTGEHGDLTGDLFIDCTGFRGLLLNQALEEPFI
SYQDTLPNDSAVALQVPMDMERRGILPCTTATAQDAGWIWTIPLTGRVGTGYVYAKDYLSP
EEAERTLREFVGPAAADVEANHIRMRIGRSRNSWVKNCVAIGLSSGFVEPLESTGIFFIHHAIE
QLVKNFPAADWNSMHRDLYNSAVSHVMDGVREFLVLHYVAAKRNDTQYWRDTKTRKIPD
SLAERIEKWKVQLPDSETVYPYYHGLPPYSYMCILLGMGGIELKPSPALALADGGAAQREFE
QIRNKTQRLTEVLPKAYDYFTQ
>TiaM
MADAMPKVIVIGGGPGGSTAAGLLAKGGVEVKLLERAEFPRYHIGESITTSCRAIIDYVGALE
KVNACGFTTKTGVLMRWGQEKDWVIDWTAQFGADTRSWQVDREIFDQVLLDNAADSGAE
VVQQAHVKRVLFDGDRAVGVEWQPAGGELRTETADYVIDASGRAGLISAQHYNNRRPHEIF
RNVAIWGYWENAELLPDSPAGGINVISSPDGWYWLIPLRDNRFSVGFVTHKTNFVERRREFD
SQEAMLAAVLGESETMKQVLAQATQLPGVRIEQDFSYAADKFCGDGYFLVGDSACFLDPLL
STGVHLAMYSGMLSAASILSLQRGEVNEPEAQAFFESLYRNAYHRLFTLVISFYQQHAGKDR
YFALADTLSRAAQGDTQSADLAFGEIIAGLTDLREAADRAGTAAAPITETIDAAATGGAKPV
QELLGAAEQARLRAEESSPNQRVTMAPMYIDANDLYDAASGLYLVTEPRLGISRVTSEAAVA
AG
>VhaH
MSVEDFDVVVAGGGPGGSTVATLVAMQGHRVLLLEKEVFPRYQIGESLLPATVHGVCRML
GVADELAGAGFPIKRGGTFRWGARPEPWTFHFGISAKMAGSTSHAYQVERARFDEILLNNAK
SKGVVVREGCSVHDVVEDGERVTGAKYTDPDGNEREVSARFVIDASGNKSRLYSKVGGSRN
YSEFFRSLALFGYFEGGKRLPEPVSGNILSVAFDNGWFWYIPLSDTLTSVGAVVRREDADKIQ
GDREKALNALIAECPLISEYLSNATRVTTGKYGELRVRKDYSYQQETYWRPGMVLVGDAAC
FVDPVFSSGVHLATYSALLAARSINSVLAGDLDEKTALNEFEARYRREYGVFYEFLVSFYQM
NVNEESYFWQAKKVTQNQSTDIESFVELIGGVSSGETALTAADRIAARSAEFAAAVDEMAGG
DGDNMVPMFKSTVVKQAMQEAGQVQMKALLGEDAEPELPLFPGGLVTSPDGMKWLPHHP
A
>VirX1
MKIESVAIVGGGSSGWMTAAALSKLCPQLEIALIEDPNIKTVGVGESTLGHFNKFLHLLDLKD
EDWMPACNATYKNSIRFTNFREGKGEVFEYPFGPSLDVSFFSQTDGINTWGKLANKYPEDFP
PETFARFVNSNTYLAEHNRLTRNKDNKIPNFNFDWDTAYHIDAELFGQYLKEKIALPNGVKH

IQGKVTGYQKESPNNHNFKYIILDQETAIFADLYIDCTGFKSLLLGEFMGEAFSPFSKKLANDK
AMATRIPYENREEEMHNVTDCHAMKNGWVWNIPLWNRIGTGYCYSSRFVSKDDAEAEFRE
HLGERGKDAKIFHIDIGHGKRTRAWVNNCVGIGLSYGFIEPLESTGLLTTHENIENLVYLINQR
DGYVTQAERDGFNYTCDHQIDSFSDFVAMHYAYSMRTDTPYWKWCTQMCNYMPESMGPH
RQKQSTWQDLSTDTIGLNTWHINHNGISFIIAGHGLRPQSYDKLSEVLLKRNNESDYYYEDIR
KDWLKHYESMVEYVKTLPTHYEFLRDEIYGSAE

>XanB1

MPAAGQAPGPECPDVLIIGGGPAGCAAAIVLAQQGWAVTLLEKEQHPRFHIGESLLPMNIPIL
ERLGALEQVRAIGVLKRGADFPNDAGGYNTFRFSHALDAKADYAFQVPRAQFDQVLFARAR
EVGVDAREQVKVESVAFDGEQPVLQARGVDGVQQFRPRYLLDASGRDTFMGNRLKLKRAN
AKHQSAAVFSHFRGVTRRPGEDAGNISIYRHAHGWMWLIPLPDDVMSVGAVCYPDYMKTR
KGDSEGFLLRTLALNPEVAARMADAERVAPVHATGNYAYECTRMSGPRWLLLGDAYTFVD
PMFSSGVFLAMHSAERGAAMVDAVLRAPQREASLQRALSRELARGLSEFKWFIYRFTSPTMR
ALFAQPQNVWQVEQAVVAMLAGDVFDSPKVRRRLRVFRAIYSVTALSMLPRAWHAWRHR
RRQARLGFDGDTLHRDAP

>Xc1333

MTQARIRKVVIAGGGTAGWVAAAALAHQFRGLLEIVLVESEQIGTIGVGESTVPPIRAFHRFL
QIDEQEFLRAVAGTFKLAISFEDWQGQGDRYIHPFGSTGQGTLVCAFHHFWLEAQRRGMHS
GFDEYCLEAQAARADRFAVLQQPAVNYAYHLDAGLYARFLRTHAQRYGATRIEGKIQQVR
QHADSGFVEALVLEDGQVVEGDLFIDCTGFRGLLIEQTLHTGYEDWSHWLPCDRAVAVQTE
SVEPPVPYTRAIAHAAGWRWQIPLQHRVGNGLVYARAHLSDDEASAMLLAAVQGTPRRDP
MLVPFVTGRRLQTWNKNVVALGLASGFVEPLESTSIHLAIVAVMRLITLFPADGIAQPQVDLF
NRISRAEAEHVRDFVILHYHATQREEALWRQCRAMELPESLQLRLRAWREGAHAWQDGDE
LFRVDSWTHVLLGQRVAPAQHHPLTRALPDQHLQQLLDGIRQPLQHVVTQLPTQQAFIDSYC
KVDQSTWG

>Xc4156

MHAPAPSESQRLVRRVVIAGGGTAGWMAAAALSKLLGRQLQITLVESDEIGTVGVGEATIPS
LVTFHRLLEIDEAQFMAATQATFKVGIAFEHWRDVDRHYIHSFGHTGTDHWSAGFQHFWLK
AHARGVARDFGDYCLELRAAQEGRFAHLPNGGMNYAYHLDAGLYARFLRRFSEGFGVQRI
EGRIGSVQTDAHSGDIAALVLDDGTRIEGDLFLDCTGFRALLIGQTLGVGSEDWSRWLFADS
ALAVQTESVGAPVTFTRARADRAGWMWRIPLQHRVGNGIVYSSRYTDQDSAAQVLEHNLQ
GRALTTPRALRFTPNQRHRVWEKNCVALGLASGFLEPIESTNIHLIQRGIVRLLQTFPQVIDPV
DIAEYNRQAAEEIAHIRDFVILHYHATDRRDTAFWRDCASMEIPDSLRHRMELFRQSGRVFH
QGNELFAENSWIQVMLGQGIVPRHHHPVADLMGDAELSQFLEGIRQRVEATLARLPPHAEFL
RRYCPAPAPPAPMPQPAPGTPLAAPAA

>Xc4345

MIPAPLRNIVIVGGGTAGWMAAAAFARVLGPSFNVQLIESEQLGTIGVGEATVPHIKAFNNLL
GIDEAEFVRQTQGSFKLGIEFVDWQRPGTSYIHGFGTQIGHPLGLLPFHQYWIKQQRRGKAQP
LGAYTLNTVAATRGKFMTSAGDVPANSPLASIAYAYHFDASQYARFLRGYAEQRGVTRLEG
LIEQVQLDAHTGSVQSVQLASGQVVSGDLFIDCSGFRGLLIEEALHTGYHDFTHWLPCDRAL
AVPCEKVGPPTPYTRSTARAAGWQWRIPLQHRTGNGYVYSSAHISDDEAAAALLANLDGTP
LADPRPLRFTTGRRKQFWNRNVVALGLASGFLEPLESTSIHLIQSGISKLLELFPREGISPVLVQ
RYNDRLAFEFDRIRDFLVLHYHATERDDSAFWRHCRTMPITPELQTTLDLFRDSGRFYRNAE
EMFAEISWVQVMVGQGILPQGYHPLVDQVPDTDAEGFLASVAQTISHCVDVMPTHQQFIDR
YCRADPVR
